# Regional habitat suitability for aquatic and terrestrial invasive plant species may expand or contract with climate change

**DOI:** 10.1007/s10530-023-03139-8

**Published:** 2023-07-28

**Authors:** Emma Nikkel, David R. Clements, Delia Anderson, Jennifer L. Williams

**Affiliations:** 1 Department of Geography, University of British Columbia, Vancouver, BC Canada; 2 Department of Biology, Trinity Western University, Langley, BC Canada; 3 Department of Geography and Biodiversity Research Centre, University of British Columbia, Vancouver, BC Canada

**Keywords:** Invasive species, Climate change, Habitat suitability models, Terrestrial, Aquatic

## Abstract

The threat of invasive species to biodiversity and ecosystem structure is exacerbated by the increasingly concerning outlook of predicted climate change and other human influences. Developing preventative management strategies for invasive plant species before they establish is crucial for effective management. To examine how climate change may impact habitat suitability, we modeled the current and future habitat suitability of two terrestrial species, *Geranium lucidum* and *Pilosella officinarum*, and two aquatic species, *Butomus umbellatus* and *Pontederia crassipes*, that are relatively new invasive plant species regionally, and are currently spreading in the Pacific Northwest (PNW, North America), an area of unique natural areas, vibrant economic activity, and increasing human population. Using North American presence records, downscaled climate variables, and human influence data, we developed an ensemble model of six algorithms to predict the potential habitat suitability under current conditions and projected climate scenarios RCP 4.5, 7.0, and 8.5 for 2050 and 2080. One terrestrial species (*P. officinarum*) showed declining habitat suitability in future climate scenarios (contracted distribution), while the other terrestrial species (*G. lucidum*) showed increased suitability over much of the region (expanded distribution overall). The two aquatic species were predicted to have only moderately increased suitability, suggesting aquatic plant species may be less impacted by climate change. Our research provides a template for regional-scale modelling of invasive species of concern, thus assisting local land managers and practitioners to inform current and future management strategies and to prioritize limited available resources for species with expanding ranges.

**Supplementary Information:**

The online version contains supplementary material available at 10.1007/s10530-023-03139-8.

## Introduction

The effects of climate change on the distributions of invasive plant species are increasingly concerning given the substantial threats of invasive species to biodiversity and ecosystem structure (Mack et al. 2000; Hellmann et al. 2008; Whitney and Gabler 2008; Crossman et al. 2011). Global climate change is predicted to critically increase invasion risk (Bradley et al. 2010), with increases in non-native species distributions predicted to be particularly strong in temperate regions (Bellard et al. 2013; Seebens et al. 2020). A study of the continental USA showed that, while 80% of current invasive plant hotspots were geographically stable with climate change, 20% are shifting northward (Allen and Bradley 2016). Not only does climate change allow for novel habitats of climatic suitability for invasive species, non-native and invasive species often have an increased ability to adapt to climate change, through their higher growth rates, wider environmental tolerances, and shortened generation times, among other traits, compared to native species (Whitney and Gabler 2008; Willis et al. 2010; Clements and DiTommaso 2011). Furthermore, climate change is allowing for increased and altered pathways for tourism and commerce, which aids the transport and spread of invasive species (Hellmann et al. 2008; Seebens et al 2015).

Identifying invasive species that could shift their distributions under the influence of climate change and other human influences provides an opportunity to target species before they can establish and spread. Many regions assess the risk of invasive species, placing medium to high-risk species that have not yet arrived (or are very recently introduced) on an ‘early detection and rapid response’ (EDRR) list. These risk assessments often do not incorporate climate change or the potential for range shifting in the future (Chai et al. 2016). A recent study of the incorporation of climate change into invasive species management showed that there is a considerable need for more targeted research, accessible science communication, and two-way dialogue between land managers and researchers (Beaury et al. 2020). The use of species distribution models is a cost-effective way to prioritize and focus actions on those species of highest invasion concern (Elith and Leathwick 2009; Bellard et al. 2013), as land managers frequently report that lack of funding and personnel limited their ability to manage invasive species (Beaury et al. 2020). Moreover, if invasive species are given the time to establish and become widespread, eradication can become nearly impossible, leaving costly containment and impact reduction strategies as the only options (Rockwell-Postel et al. 2020). The use of habitat suitability models (HSMs) and EDRR lists can work in tandem to account for shifting invasive species and the risks posed by their spread.

Habitat suitability models (alternatively known as ecological niche, envelope, or species distribution models, depending on the application) are used as tools to predict the current potential suitability of an area to invasive species (De Kort et al. 2020). Habitat suitability models (HSMs) develop correlative relationships between species location or occurrence data and the environmental or climatic conditions in which those occurrence points are found (Peterson 2003; Cordier et al. 2020). These models produce predictive approximations of an area’s current suitability and can be used to project these responses into future climate scenarios or alternate locations. HSMs are widely used in reserve planning for conservation, predicting extinctions or extirpations of species under future climates, and predicting species invasion risk (Bocsi et al. 2016; Barbet-Massin et al. 2018). While predictive outcomes are known to be variable across model types (Qiao et al. 2015; Hao et al. 2019), an ensemble modelling approach that results in a consensus model from multiple individual model algorithms improves the reliability of habitat suitability predictions (Marmion et al. 2009; Hao et al. 2019; Čengić et al. 2020). The use of HSMs can play a vital role in our ability to identify areas of high suitability for invasive species before they become established.

Habitat suitability analyses are often conducted at a global or continental scale, but there is a need for regional assessments where results can be more readily interpreted and applied by managers (Gervais et al. 2020; Lathrop et al. 2018). While risk assessments have been completed on many species with invasive potential in the Pacific Northwest region of North America (PNW), they are often completed without an assessment of the impacts of climate change (Gervais et al. 2020). The PNW is currently considered an invasion hotspot and invasions are predicted to increase with climate change (Bellard et al 2016); however, the impacts of climate change on particular invasive plants are generally unknown. The region is increasingly susceptible to the spread of invasive species due to its location in a temperate zone, its position as highly trafficked ports and tourist destinations, and its vastly heterogeneous landscapes and changing land-use. In a recent review of the predicted effects of climate change on invasive species present or considered an invasion threat to the PNW, only six studies focused specifically on the expansion or abundance of invasive species due to climate change within the PNW region, none of which included aquatic plants or terrestrial plants of coastal PNW (Gervais et al. 2020). Thus, to prioritize the monitoring of species and establishing preventative strategies, a more detailed understanding of the current and future potential habitat suitability of invasive plant species in the PNW is necessary.

For this study, we selected four species with varying habitat requirements, occupied niche space, and invasion status from the EDRR list for British Columbia (BC), Canada. Two terrestrial species, *Geranium lucidum* and *Pilosella officinarum*, and two aquatic species, *Butomus umbellatus* and *Pontederia crassipes*, were chosen based on their limited establishment in the PNW, the potential risk they pose, and the major impacts they have elsewhere in their introduced ranges. *Geranium lucidum* poses a high risk to sensitive woodland habitats as seen by its establishment as an invasive species in Oregon, USA (Dennehy et al. 2011), while *Pilosella officinarum* displaces native species in temperate and sub-alpine climates of its invaded range, including Argentina, New Zealand, and the USA (CABI 2022b). The tropical and sub-tropical *Pontederia crassipes* has spread from its introduction as an ornamental to become one of the worst aquatic invasive plants globally (Villamagna and Murphy 2010), in contrast to *Butomus umbellatus*, which is currently only invasive in North America, but is tolerant of a wide range of temperatures and quickly establishes itself along disturbed freshwater shorelines (Cao et al. 2022). Using habitat suitability models, we aimed (1) to establish the current potential habitat suitability of four relatively new invasive plant species to the PNW region of North America, assessing the relative contributions of climate and human influence, and (2) to predict the future habitat suitability for these species in the PNW, assessing the potential expansion or contraction of the distribution of these species with climate change, and comparing and contrasting species from different habitat types.

## Methods

### Study species

We selected four focal species from the British Columbia provincial EDRR (early detection and rapid response) list (Early detection and rapid response 2022), in conjunction with consultation with invasive plant experts from the Invasive Species Council of Metro Vancouver and the Provincial government of BC. Species placed on the EDRR list are identified as not currently present, or present in a limited extent, in BC and assessed as posing a high or medium risk, prompting the development of a response plan with the goal of eradication (IMISWG 2014). These species likewise pose a threat to other areas of the Pacific Northwest, particularly the areas west of the Cascade Mountain Range in Washington state (WA) and Oregon (OR).

*Geranium lucidum* L. (Geraniaceae), or shiny geranium (hereafter referred to as *G. lucidum*), is an annual herbaceous terrestrial plant originating from Europe and temperate Asia (USDA 2013). *Geranium lucidum* is considered invasive in Australia, New Zealand, USA, and Canada, and was first collected in North America in 1971, in Oregon (Dennehy et al. 2011). *Geranium lucidum* forms dense mats, spreading by seeds that germinate from February to October, leading to up to 5 generations in a single growing season and creating a persistent seed bank (USDA 2013).

*Pilosella officinarum* Vaill. (Asteraceae), syn. *Hieracium pilosella*, or mouse-ear hawkweed (hereafter referred to as *P. officinarum*), is a perennial herbaceous terrestrial plant, originating from temperate and sub-arctic Europe (Bishop and Davy 1994). *Pilosella officinarum* is considered invasive in similar climates of New Zealand, Australia, Argentina, USA, and Canada. Its introduction date and pathway in North America is unknown; however, it likely spread from ornamental plantings or as a contaminate of agricultural pasture seed (CABI 2022b). *Pilosella officinarum* can spread via seed, which is often wind-dispersed, but more often spreads vegetatively by producing daughter rosettes from stolons that spread rapidly and create dense mats (Bishop and Davy 1994).

*Butomus umbellatus* L. (Butomaceae), or flowering rush (hereafter referred to as *B. umbellatus*), is a sedge-like perennial aquatic plant, originating from Eurasia (Anderson et al. 1974) and considered invasive in the USA and Canada. *Butomus umbellatus* was first recorded in North America along the St. Lawrence River near Montreal in 1897 and has since spread throughout the Great Lakes region of eastern North America as well as northwestern USA and western Canada (Anderson et al. 1974; Gaskin et al. 2021). *Butomus umbellatus* can grow as an emergent plant in shallow waters (< 3 m) or as a submerged plant in deeper waters (3–6 m) (Jacobs et al. 2011). It establishes quickly in disturbed areas or areas of sparse aquatic vegetation, with fluctuating water levels promoting establishment and dispersal, and is tolerant of a wide range of temperatures (Cao et al. 2022).

*Pontederia crassipes* (Mart.) Solms, formerly *Eichhornia crassipes*, (Pontederiaceae), or common water hyacinth (hereafter referred to as *P. crassipes*), is a tropical aquatic plant, originating from the Amazon basin of Brazil (CABI 2022a). *Pontederia crassipes* has spread to nearly all tropical and subtropical regions of the globe and has been found seasonally in higher latitudes. Imported as an ornamental pond plant, *P. crassipes* was introduced to North America in 1884 for an exposition in New Orleans (USDA 2020). As a free-floating, freshwater plant, *P. crassipes* forms dense mats of vegetation that spread horizontally in uncrowded, shallow waters but can elongate to 1 m in height in crowded, deeper waters (Villamagna and Murphy 2010), where it can spread vegetatively or through seeds which are released below water and may remain dormant in mud for many years (CABI 2022a).

### Species data

**Fig. 1:**
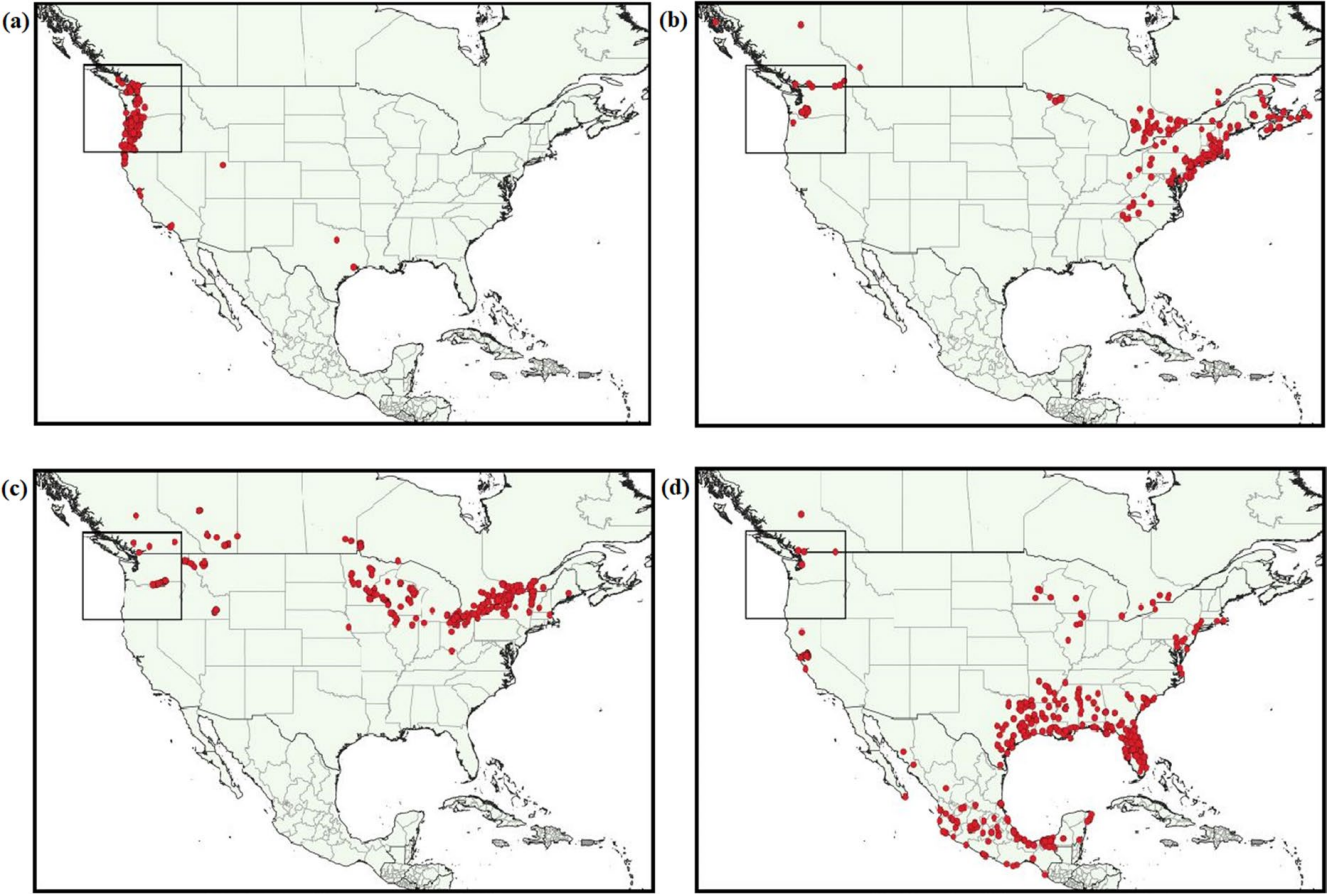
Species presence record locations after data cleaning for **a** *Geranium lucidum*; **b** *Pilosella officinarum*; **c** *Butomus umbellatus*; and **d** *Pontederia crassipes*. Inset box shows the PNW study region

Species occurrences were collected from three internet databases: Global Biodiversity Information Facility (GBIF), Early Detection and Distribution Mapping System (EDDMapS), and Invasive Alien Plant Program (IAPP) (Data citations available in Table S1.1) (EDDMapS 2022; GBIF 2022; IAPP 2022). The combination of invasive and native range data has been used in a number of studies under the assumption that occurrence data from the species’ native region is necessary to account for the fundamental niche of the species; however, several studies have shown that the realized niche of a species in its invaded range can differ significantly from its native range (Sittaro et al. 2023). The result of using both the native and invasive range of a species puts SDMs at risk of overestimating niche overlap (Broennimann et al. 2011; Zhang et al. 2020), suggesting that the transferability of niche space is often limited between different geographical regions (Duque-Lazo et al. 2016). Additionally, invaded ranges often encompass both the climatic range of the native distribution as well as potential novel conditions (Barbet-Massin et al. 2018). Therefore, we chose to include species records from the species’ invaded range in North America only. Whereas species record and environmental variable data for the entirety of North America was used to develop the models, mapped results presented here include only BC, WA, and OR, as the general circulation models we used were focused on, and thus most appropriate, for western North America (Mahony et al. 2022). To ensure the species records correspond with the available environmental variables, records before 1980 were removed. To account for spatial bias and avoid model over-fit, only one record per km^2^ was retained, chosen according to quality of the data source, as non-governmental organizations, academic institutions, or government agencies are of higher quality than records sourced from community science, such as iNaturalist. Additionally, occurrence records underwent significant cleaning to keep records with coordinate uncertainty of < 1000 m and complete time and place data, while removing duplicate records, country centroid coordinates, or records with inconsistencies in time or place of recording. After filtering and cleaning the occurrence record, 611 records remained for *G. lucidum*, 241 records for *P. officinarum*, 524 records for *B. umbellatus*, and 654 records for *P. crassipes* (Fig. 1). All filtering and cleaning of occurrence records was done using R 4.1.1 (R Core Team 2021), using additional packages ‘CoordinateCleaner’ (version 2.0.18; Zizka et al 2019) and ‘dplyr’ (version 1.0.6; Wickham et al. 2021). Detailed methodology and R scripts for all data cleaning and species modelling is available in a public GitHub repository accessible at https://github.com/enikkel/PNW-Habitat-Suitability-Modelling.

### Environmental data

To account for variables influencing the distribution of these invasive plant species, we considered both bioclimatic variables and a human influence index (Table S1.2). We used 33 bioclimatic variables available from ClimateNA (AdaptWest Project 2021), averaged for the 1981–2010 period, at a 30 arc-second resolution (approximately 1 km^2^). These variables consist of biologically relevant means and indices, including seasonal and annual means, extremes, growing and chilling degree days, and drought indices (Wang et al. 2016). We used the Human Influence Index provided by NASA’s Socioeconomic Data and Applications Center (SEDAC) to provide a geographic projection of the anthropogenic impacts on the environment, at 30 arc-second spatial resolution, created from layers including human population density, human land use and infrastructure, and human access (such as roads, railways, or coastlines) (WCS and CIESIN 2005). We initially considered a subset of land cover variables in addition to climate and human influence factors; however, this inclusion resulted in either no effect on the models (terrestrial species) or a masking effect which reduced the contributions of all other variables (aquatic species). Thus, land cover variables were excluded from use as model predictor variables.

For future climate projections, we considered three representative concentration pathways (RCPs), also corresponding to shared socioeconomic pathways (SSPs), from the 6th assessment report from the Intergovernmental Panel on Climate Change (IPCC 2021). While RCPs convey differing amounts of greenhouse gas concentrations, SSPs couple these projections with varying levels of actions addressing climate change through factors such as population, technological advancements, and/or economic growth (Riahi et al. 2017). To address these varying pathways for future climate, we considered RCP 4.5 (SSP2), RCP 7.0 (SSP4) and RCP 8.5 (SSP5) for the model projections. While CO_2_ levels under RCP 4.5 correspond to the current ‘middle of the road’ scenario, RCP 7.0 and RCP 8.5 follow increasing levels of CO_2_ in progressively ‘worse’ scenarios (Riahi et al. 2017). The top three general circulation models (GCMs) for the Western North American region were used to account for the spatial variation in climate change responses (Mahony et al. 2022). Averaged projections for 2050 (2041–2060) and 2080 (2061–2090) were used from GCMs MRI-ESM2.0 (MRI), UKESMI1.0-LL (UK), and MPI-ESMI1.2-HR (MPI) (Table S1.3).

### Modelling methods

To predict the potential habitat suitability of each of the four species in the PNW, we performed habitat suitability modelling and ensemble forecasting using the ‘biomod2’ package (version 3.5.1; Thuiller et al. 2009) with R 4.1.1 (R Core Team 2021). For each species, we used six of the algorithms most commonly used for HSMs (Hao et al. 2019) including: three regression methods, (1) Generalized Linear Model (GLM, McCullagh and Nelder 1989), (2) Generalized Additive Model (GAM, Hastie and Tibshirani 1990), and (3) Multivariate Adaptive Regression Splines (MARS, Friedman 1991); and three machine learning methods (4) Random Forests (RF, Breiman 2001), (5) Generalized Boosted Model (GBM, Ridgeway 1999), and (6) Artificial Neural Network (ANN, Ripley 2007). To predict habitat suitability, presence and absence data should be used; however, when no true absence data are available, pseudo-absence data must be generated (Čengić et al. 2020). Ensemble modelling requires that the model accuracy be compared to determine which models to include, therefore the same data must be used by all algorithms to remain unbiased (Hao et al. 2019). Thus, we generated the same number of pseudo-absences as presence records, ran pseudo-absence generation 10 times (Barbet-Massin et al. 2012), and randomly selected the pseudo-absences from within a geographic extent based on the species in question (according to the methods described by VanDerWal et al. 2009). For each species, test models were performed at 100 km intervals (from 100 to 500 km from the presence record) to determine the appropriate maximum distance for pseudo-absence selection, based on model evaluation statistics and the number of variables contributing to the model (the number of contributing variables reducing from 3 + to 1–2 variables suggested the distance was too great). Therefore, pseudo-absences were selected from within a minimum 1 km distance from presence records and a maximum 200 km distance from *G. lucidum* presence records, 500 km from *P. officinarum* presences, 400 km from *B. umbellatus* presences, and 400 km from *P. crassipes* presences.

To calibrate and test the models, 70% of the species records were randomly selected as training data, and the other 30% were used as testing data. We used two evaluation metrics: the area under the relative operating characteristic curve (AUC, Fielding and Bell 1997) and the true skill statistic (TSS, Allouche et al. 2006), to assess the model's ability to discriminate between an area of presence or absence. We repeated the cross-validation and evaluation operations five times to obtain an average value of model performance. To identify collinearity between variables, correlations between all 34 variables (Table S2.1) were assessed by calculating a variance inflation factor (VIF) for each variable (R package ‘usdm’ version 1.1.18; Naimi et al. 2014). Variables were retained for the HSMs if they had a VIF of less than 5 (Tables S2.2–2.5). This process resulted in seven variables retained for *G. lucidum*, *P. officinarum*, and *B. umbellatus*, and eight for *P. crassipes*. We assessed contributions of each variable to each species’ model through the ‘variable importance’ procedure in the ‘biomod2’ package (Thuiller et al. 2009). This procedure was repeated three times for each variable, finding the mean correlation coefficient over all cross-validation runs, resulting in a ranking of variable importance for each model. For each individual model and the final ensemble model, we evaluated the response of the species to environmental predictor variables with the evaluation strip method (Elith et al. 2005), to assess how each variable contributed to the model.

For a robust forecast of current and future habitat suitability, we used ensemble forecasting to combine the six modelling algorithms (Araujo and New 2006, Thuiller et al. 2009). Models with a TSS > 0.7 were included in the ensemble model. To produce the final current climate ensemble model, we used the mean weighted by each model’s TSS value, rather than equally weighting the mean of all models, as it has been found to perform better (Marmion et al 2009). Overall, a total of 300 projections of habitat suitability (6 modelling algorithms × 10 pseudo-absence runs × 5 cross-validation runs) were created for each species. One current climate habitat suitability map and three future climate habitat suitability maps per year (2050 and 2080) and per scenario (RCP 4.5, 7.0 and 8.5) were produced per species, after future habitat suitability maps were averaged per year and per scenario. These maps contain continuous probabilities of species occurrence, transformed into integers from 0 to 1000 by the biomod2 functions, in order to convey the relative suitability of an area rather than generating a threshold of ‘unsuitable’ vs ‘suitable’ habitat. Additionally, to assess the extent of extrapolation and environmental similarity between the climates where the species records were located and the PNW study region, we performed a Multivariate Environmental Similarity Surfaces (MESS) analysis. The MESS analysis can indicate novel predicted environments which may be regions where caution is needed when interpreting the model results (Elith et al. 2010). All map visualizations were done in QGIS (version 3.22.3 Białowieża, QGIS Development Team 2022). The ODMAP protocol (Zurell et al. 2020) was used to outline the main steps involved in building the SDMs and can be found in the Supplemental Materials.

## Results

### Model evaluations

Overall, most model iterations performed well with an average TSS value of 0.728 and an average AUC value 0.918, across all species (Table 1). Response curves generated for each predictor variable showed moderate to strong responses to climate variables across species. Response curves can be viewed as the likelihood of presence or of suitable habitat, compared to random environmental space, rather than ‘probability of presence,’ because the training and testing data contains pseudo-absence data rather than true absence data. For all four species, the most limiting variables on the likelihood of a species presence were bioclimatic factors, particularly temperature related variables (Table 2). Additionally, the response curves of all species showed an increase in the likelihood of suitable habitat with increasing human influence index value. Furthermore, the results of the Multivariate Environmental Similarity Surfaces (MESS) analysis showed that the PNW climate data is reasonably similar to the climate data of the species record locations (Fig. S4.1), suggesting that our models are not extrapolating beyond known data.

**Table 1:** Habitat suitability model evaluation metrics: AUC (area under the relative operating characteristic curve) and TSS (true skill statistic). 300 projections were run for each species; final ensemble models include only those with TSS > 0.7

Species	Mean values (all 300 models)		Ensemble modelling values		Number of models included in ensemble
	AUC	TSS	AUC	TSS	
*Geranium lucidum*	0.96	0.85	0.98	0.87	299
*Pilosella officinarum*	0.88	0.65	0.95	0.74	67
*Butomus umbellatus*	0.91	0.70	0.98	0.85	154
*Pontederia crassipes*	0.92	0.71	0.96	0.77	184

**Table 2:** Effect of selected environmental variables on current likelihood of potential habitat based on the response curves (Figs. S3.2, S3.4, S3.6, S3.8). A variable was considered to have a slightly limiting effect when the response was between 0.5 and 0.75 (likelihood of presence) for some values of the variables and a limiting effect when the species response fell below 0.5. Responses showing an increase in the likelihood of presence with increasing variable values is noted as positive, while responses showing a decrease in the likelihood of presence with increasing variable values is noted as negative. (All values are approximate and show the upper and/or lower range value in the units of the associated variable, as determined by the species response curves.)

Variable	*Geranium lucidum*	*Pilosella officinarum*	*Butomus umbellatus*	*Pontederia crassipes*
Annual heat-moisture index	–	Limiting > 20	–	–
Summer heat-moisture index	Slightly limiting (negative)	–	–	Limiting < 1200 and > 1400
Degree-days below 0 °C (chilling degree-days)	Limiting > 125	Limiting > 750 degree-days	Limiting < 300 and > 1700 degree-days	–
Degree-days above 18 °C (cooling degree-days)	Slightly limiting (negative)	–	–	–
Number of frost-free days	–	–	–	Slightly limiting (positive)
Day of the year the frost-free period begins	–	Slightly limiting < 120 and > 150	–	–
Precipitation as snow (mm)	–	–	Limiting > 200 mm	Limiting (negative)
Extreme maximum temperature over 30 years	–	–	Limiting < 34	–
Relative humidity (%)	Slightly limiting < 60 and > 75%	–	Limiting < 53%	Slightly limiting (positive)
Summer (June, July, and August) precipitation (mm)	Limiting > 150 mm	–	–	–
Winter (December, January, February) precipitation (mm)	–	–	–	–

### Current potential habitat suitability in the Pacific Northwest region

**Fig. 2:**
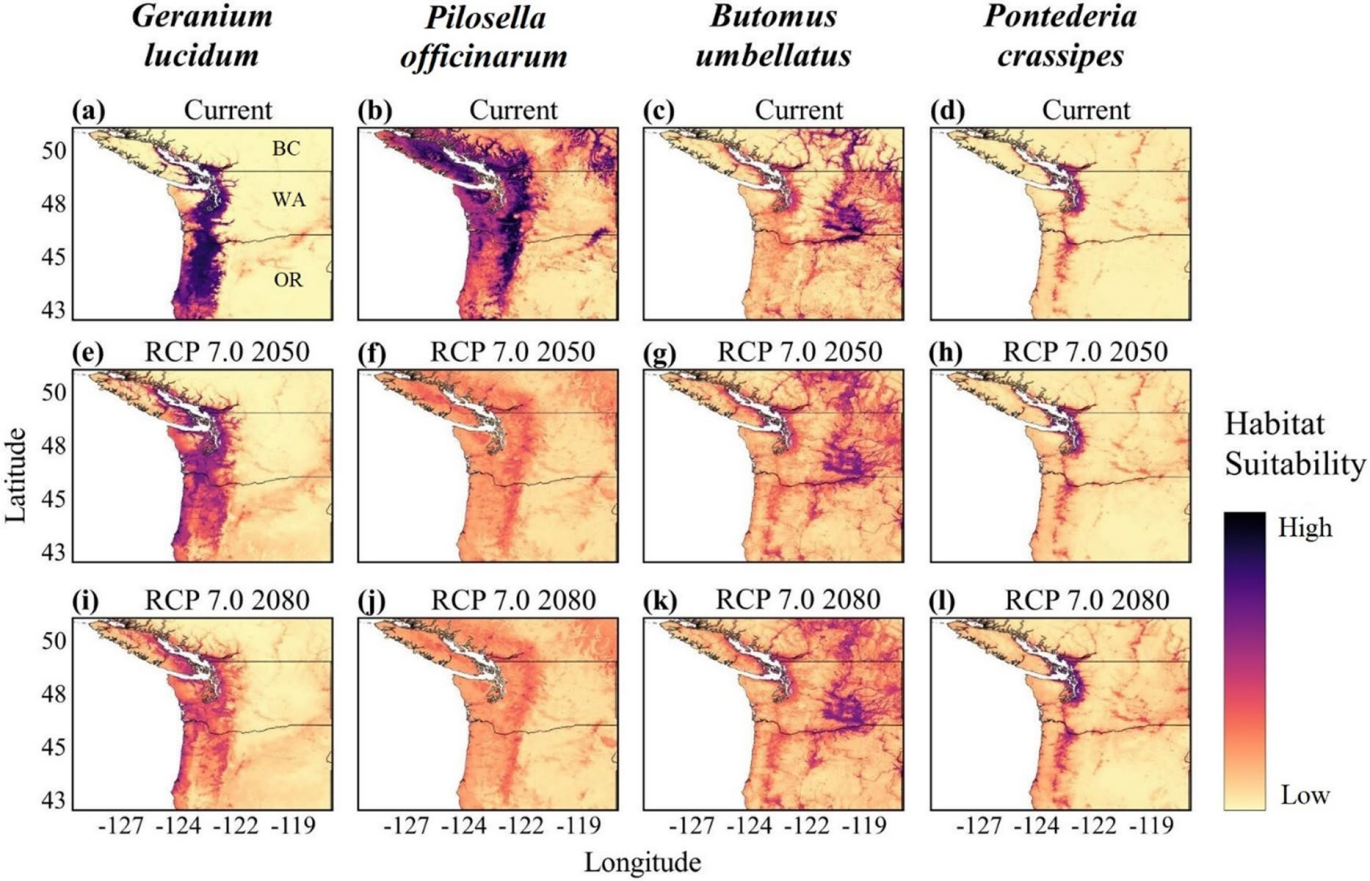
Current and future projected habitat suitability in PNW for the current potential habitat suitability (**a**–**d**), RCP 7.0 (2050) scenario (**e**–**h**), and RCP 7.0 (2080) scenario (**i**–**l**) for *Geranium lucidum*, *Pilosella officinarum*, *Butomus umbellatus*, and *Pontederia crassipes*, respectively. Pacific Northwest regions include British Columbia (BC), Canada, Washington State (WA), USA, and Oregon (OR), USA, as noted in panel 2a

The current range of habitat suitability for the terrestrial species *G. lucidum* is predominantly in the sheltered coastal and valley regions of the PNW, west of the Cascade Mountain Range and east of the coastline, where suitability declines with increasing elevation (Fig. 2a). The variable importance procedure ranked degree-days below 0 °C, degree-days above 18 °C, and summer precipitation as the top three bioclimatic variables contributing to this model (Fig. S3.1). Furthermore, these variables are also the most limiting (Table 2) with decreased likelihood of presence with increasing number of days below 0 °C, increasing number of days above 18 °C, and increasing summer precipitation.

The current range of habitat suitability for the terrestrial *P. officinarum* is found along the Cascade Mountain Range through OR, WA, and south-west BC, as well as in the higher elevations of Olympic National Park and the majority of central to eastern Vancouver Island (Fig. 2b). The variable importance procedure ranked degree-days below 0 °C, annual heat moisture index, and the day of the year the frost-free period begins as the top three bioclimatic variables (Fig. S3.3). Additionally, unlike its lower contribution to *G. lucidum*, the human influence index contributed nearly as much as the bioclimatic variables did for *P. officinarum*, suggesting an increase in likelihood of presence in climatically suitable regions with high human activity.

The current range of potential habitat suitability for the aquatic invasive species *B. umbellatus* is found further inland, compared to *G. lucidum* and *P. officinarum*, in regions experiencing increased continental climates (Fig. 2c). These regions are predominately east of the Cascade Mountain Range, although moderate suitability is predicted in sheltered coastal locations around the Seattle and Vancouver areas. The regions with highest suitability have limited summer and winter precipitation and are subject to more extreme maximum temperatures (Table 2). The variable importance procedure ranked extreme maximum temperature over 30 years, relative humidity, and summer precipitation as the top three bioclimatic variables contributing to this model (Fig. S3.5). While increased summer precipitation is limiting, increased relative humidity and extreme maximum temperatures result in greater likelihood of *B. umbellatus* presence. In addition, increased human influence increases the likelihood of species presence.

The current range of potential habitat suitability for *P. crassipes*, the second aquatic species, is found along sheltered inland regions west of the Cascade Mountain Range (Fig. 2d). These regions reflect the most moderate temperature and precipitation areas of temperate PNW, with very low suitability predicted elsewhere in the PNW. The number of frost-free days contributed the most to limiting suitability based on variable importance; however, there is an approximately 50% likelihood that *P. crassipes* is present when the number of frost-free days is greater than 350 (Fig. S3.8). Moreover, the likelihood of its presence drops substantially with any precipitation as snow and with increased winter precipitation. The variable importance procedure ranked these three variables as the top bioclimatic variables contributing to this model (Fig. S3.7). While the response curve shows these variables to be limiting, the highest likelihood of presence under any variable is considerably lower for *G. lucidum*, *P. officinarum*, or *B. umbellatus.*

### Future potential habitat suitability

**Fig. 3:**
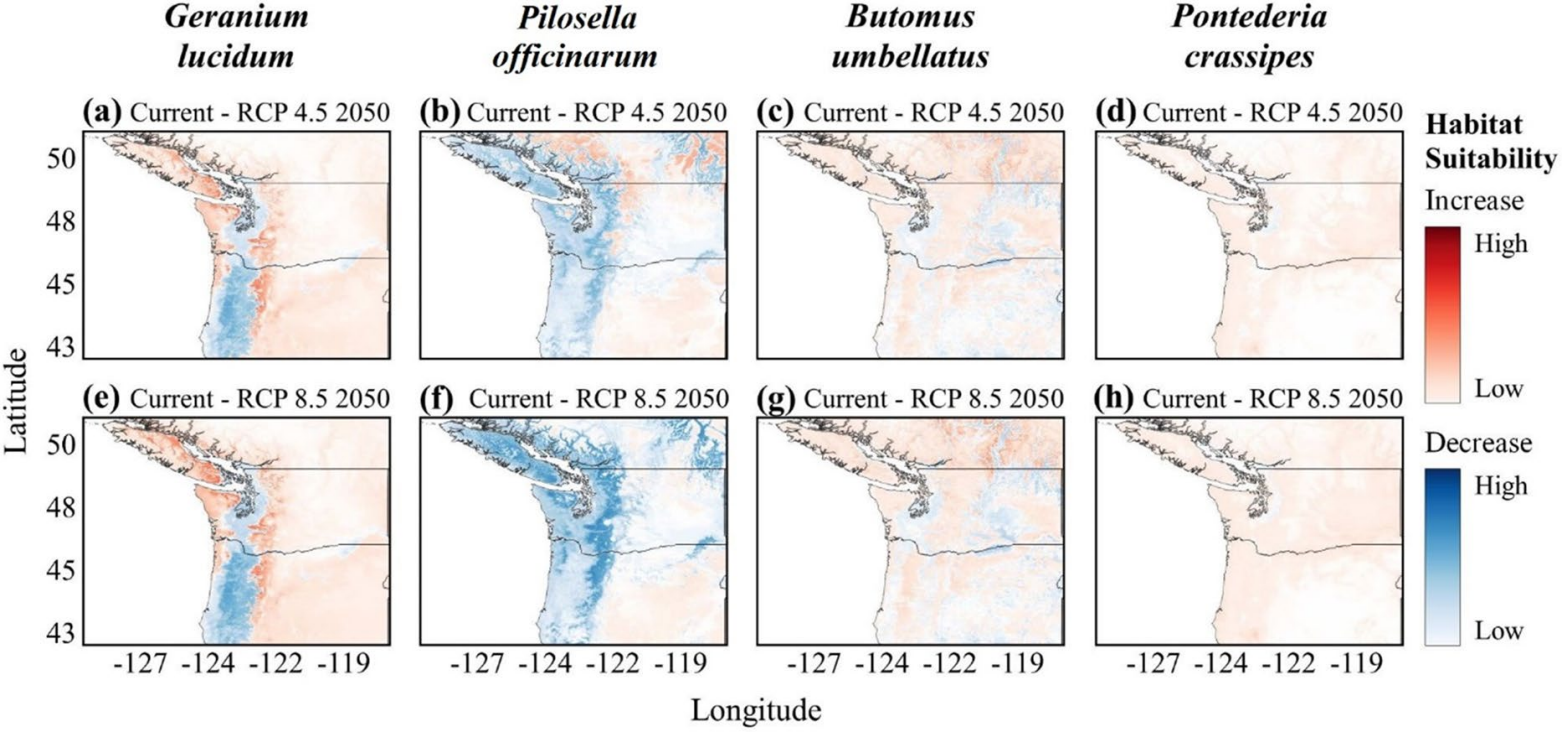
Potential expansion or contraction of habitat suitability under future climate scenarios RCP 4.5 2050 (**a**–**d**) and RCP 8.5 2050 (**e**–**h**) for *Geranium lucidum*, *Pilosella officinarum*, *Butomus umbellatus*, and *Pontederia crassipes*, respectively

Future predictions in climate change differed substantially across species. Overall, the majority of coastal PNW remains suitable for *G. lucidum* regardless of climate scenario or timeline. With climate change, *G. lucidum* habitat suitability is predicted to increase in higher elevations and higher latitudes, following the coastline north, under the ‘middle of the road’ scenario RCP 4.5 (see the Methods section for a description of the representative concentration pathway scenarios). Inland southern latitudes show a decrease in suitability by both 2050 and 2080, although most areas remain moderately suitable (Fig. 3a; Fig. S5.3). Likewise, the higher CO_2_ climate scenario RCP 7.0 leads to a more significant increase in suitability at higher coastal elevations by 2050, with coastal regions and Vancouver Island increasing from low to moderate or high suitability by 2080 (Fig. 2e and i). Conversely, a greater loss in suitability by 2080 is seen at inland southern latitudes (Fig. 2i). Habitat suitability under climate scenario RCP 8.5 follows a similar trend to RCP 7.0 (Fig. S5.2). The increase in coastal suitability toward northern regions and in higher elevations coincides with decreased suitability at the current southern limits of the range.

In contrast to *G. lucidum*, *P. officinarum* habitat suitability under climate scenario RCP 4.5 for 2050 shows a substantial decrease in lower elevations; however, the latitudinal range has low or moderate changes (Fig. 3b). Low or moderate increases in suitability are found at higher elevations and in higher latitudes. Additionally, coastal regions lose potential high suitability, but retain low suitability. Suitability under RCP 7.0 by 2050 and 2080 predicts higher decreases in suitability, particularly along coastlines and higher elevations in Olympic National Park in WA, and on Vancouver Island (Fig. 2f and j). Similarly, *P. officinarum* suitability under higher CO_2_ scenario RCP 8.5 for 2050 and 2080 suggests an overall decrease in all regions of the PNW (Fig. S5.2). Reductions in potential habitat suitability by 2080 are substantially higher under RCP 8.5 than RCP 4.5 from the Cascade Mountain Range west; however, some areas east of the Cascades may retain low suitability by 2080 under RCP 8.5 (Fig. S5.3).

For the aquatic species, *B. umbellatus* and *P. crassipes*, future climate scenarios predict far fewer substantial changes in habitat suitability compared to the terrestrial species, *G. lucidum* or *P. officinarum*. Some potential habitat suitability for *B. umbellatus* in the southern regions of the PNW is predicted to be lost under RCP 4.5 and 8.5 by 2050, while increases in suitability are predicted in the interior of BC (Fig. 3c and g). Likewise, under RCP 7.0, the extent of low and moderate suitability increases by 2050 and 2080 with some potential decreases in southern latitudes (Fig. 2g and k). Overall, regions of *B. umbellatus* habitat suitability in WA and BC will potentially increase to moderate suitability under all future climate scenarios, with some potential habitat suitability losses occurring in OR. Similarly, future climate scenarios do not predict increases to high suitability for *P. crassipes* in the PNW. Regions of moderate or high suitability under current climate are predicted to remain moderate or highly suitable under all future scenarios (Fig. 2h and l; Fig. S5.1 and S5.2). Areas surrounding regions of moderate or high suitability under current climate (Fig. 2d) are predicted to increase to low or moderate suitability under climate scenarios 4.5, 7.0, and 8.5 (Figs. 2h, l and 3d, h). Any gains or losses in habitat suitability predicted under scenarios RCP 4.5 or 8.5 by 2050 are minimal and do not suggest any change to the likelihood of suitable habitat for *P. crassipes* (Fig. 3d and h).

## Discussion

In this study, we examined potential changes in habitat suitability driven by climate change within the Pacific Northwest region of North America for four invasive plant species which have caused economic and ecological impacts in introduced regions but are not yet fully established in all areas of this region: *Geranium lucidum*, *Pilosella officinarum*, *Butomus umbellatus*, and *Pontederia crassipes*. Our results highlight the impact of climate change on invasive plants in the PNW and the variability in response across species. Across the four focal study species, we found that the range of suitable habitat available to each species is not impacted by climate change equally, as some species ranges are predicted to expand or contract, while others may shift in elevation or latitude. Further, the terrestrial species we examined were predicted to have more significant changes in habitat suitability due to climate change compared to the aquatic species.

### Impacts of climate change and human influence

The impacts of climate change and human influence factors on terrestrial species *G. lucidum* and *P. officinarum* differed greatly within the PNW. Our results suggest that the impacts of climate change will lead to increases in habitat suitability, with many areas of low to moderate suitability under current conditions becoming more suitable, especially in higher latitudes and elevations. At the same time, climate change is predicted to only moderately reduce the suitable habitat available for *G. lucidum*. Even after reductions in suitability in the currently highly suitable regions of Oregon, moderate suitability will remain and may not reduce the growth of already established populations. This predicted trend is consistent with the biology of the species and its interactions with anthropogenic factors, as it is able to form dense mats in disturbed forested areas in climates similar to its native habitat in Europe and temperate Asia (USDA 2013). Conversely, our results suggest that climate change will reduce the area of suitable habitat for *P. officinarum*, relegating it to the higher elevations and latitudes by 2050 and losing most suitability by 2080. Given the origin of *P. officinarum* in cooler areas of subarctic or temperate Europe (Bishop and Davy 1994), it makes sense that warmer temperatures would diminish its range. Potentially suitable habitats in higher elevations that *P. officinarum* has yet to occupy would benefit from increased monitoring and implementation of preventative measures. While the highly populated and travelled areas of lower elevation in the PNW may not be as vulnerable to invasion by *P. officinarum* in the long term, many parks or natural areas located at higher elevations in the PNW attract visitors to the region, potentially increasing pathways of spread to these climatically suitable areas. Furthermore, our results suggest that human influence on landscapes may have a greater impact on the likelihood of *P. officinarum* presence compared to *G. lucidum*. Our results found the only variable to have a limiting effect on *G. lucidum* to be degree-days below 0 °C. This may contribute to the limiting of *G. lucidum* in other regions, but climate change scenarios do not predict longer periods of temperatures below 0 °C in the PNW (Mote and Salathé 2010), thus leaving current populations relatively unaffected. However, unlike *G. lucidum*, *P. officinarum* requires more chilling degree-days and lower annual heat moisture, consistent with its alpine and subalpine native range.

While few habitat suitability studies have been conducted on *G. lucidum* or *P. officinarum*, previous model predictions are consistent with our results, although at a much larger spatial scale. A USDA weed risk assessment based on three climatic variables estimated that 54% of the United States and 4% of Canada is suitable for the establishment of *G. lucidum*; however, they suggest this estimate is conservative and other variables may limit the suitable range (USDA 2013). Our study of *G. lucidum* used 7 variables, covering bioclimatic and human influence factors, and while suggesting a more refined area of suitability, the overall results are consistent within the PNW region. While few studies have focused on the invasive nature of *G. lucidum*, *P. officinarum* has become a serious and widespread invasive plant in New Zealand and Argentina, prompting climate modelling in adjacent regions where the species has not become so firmly established yet (Beaumont et al. 2009). Consistent with our model, climate modelling of hawkweed species in Australia predicted that the climatically suitable habitat available to *P. officinarum* will decline overall, with some sub-alpine and alpine areas remaining climatically suitable until 2070 (Beaumont et al. 2009). Large-scale modelling studies are necessary for analysis of large-scale patterns and trends, but regional models are crucial for advising local- and regional-scale management strategies for every-day conservation or land planning efforts.

While the impact of climate on the terrestrial species examined differs, the changes in the habitat suitability in the PNW for these species are potentially more dramatic than for the aquatic species we studied. Nevertheless, freshwater ecosystems are particularly vulnerable to invasion due to a greater number of introduction pathways and heavy impacts by a variety of human activities (Havel et al. 2015; Rodríguez-Merino et al. 2018; Gervais et al. 2020), and it is critical to know which species are more likely to increase with climate change. Our results suggest that the interior of BC and Washington have a much higher potential habitat suitability for *B. umbellatus* than coastal regions. Regions of higher elevation remain mostly unsuitable; however, the likelihood of increased suitability is predicted to be stronger in higher latitudes, especially by 2080. Additionally, *B. umbellatus* may have differing climate tolerances depending on the ploidy level for a particular population, and further studies are needed to determine how levels of ploidy affect *B. umbellatus*, as seen in other invasive plant species (Clements and Jones 2021). The populations of *B. umbellatus* in invaded western North American regions are generally triploids, which are distinct from the earlier invaded eastern North American regions where the majority of populations are diploid (Gaskin et al. 2021). Recent studies have suggested that polyploids exhibit higher invasive capacity and may have a greater tolerance to increasing temperatures and rainfall (Gaskin et al. 2021; Moura et al. 2021), suggesting that projections here may be underestimates of future habitat suitability. For both *B. umbellatus* and *P. crassipes,* the importance of the human influence index on the models suggests a greater impact of human activities on aquatic species, relative to the terrestrial species. On the other hand, as a tropical species, the potential habitat suitability for *P. crassipes* under current climates is less than that of *B. umbellatus*, as suggested by the lower likelihood of presence in the variable response curve. Future climate scenarios suggest some low increases in suitability, but our results suggest that, regardless of scenario, climate change is not likely to increase the habitat suitability of *P. crassipes* substantially by 2050 or 2080. The most moderate climate areas within the PNW are predicted to have some potential suitability; however, this does not necessarily suggest an adequate climate for established populations. For example, *P. crassipes* was discovered at a location in the Metro Vancouver region in 2020 where it persisted over winter, but it did not survive over the winter of 2021 (Becky Brown personal communication 2022). Warm temperatures, and the lack of frost-days, is known to be one of the main determining factors in the growth of *P. crassipes* (Kriticos and Brunel 2016; Dersseh et al. 2019). Additionally, in a study of the *P. crassipes* infestation of Lake Victoria, Kenya, seasons of moderate rainfall and stable temperatures resulted in rapid blooming and the formation of mats (Ouma et al. 2007). In years of few frost days and very limited snow, our model predicts that the sheltered, mild-climate, inland regions surrounding Vancouver, Seattle, and Portland have the highest potential for suitable habitat although this result also suggests a correlation to human influences.

In response to the increased awareness of aquatic ecosystem vulnerability, recent studies have focused on aquatic invasive plants and their response to changing climate. In a recent study on *B. umbellatus* in North America, it was predicted that an overall decline in distribution may occur under future climate; however, there are currently several regions of potential suitability for *B. umbellatus* where no occurrences have been recorded (Banerjee et al. 2020). Banerjee et al. (2020) suggests that *B. umbellatus* may have undergone a shift in its realized climatic niche and has adapted to environmental conditions in its invasive range. Our predictions for potential suitability changes under future climate scenarios in the PNW are consistent with the suitability findings of this study, suggesting that the majority of the PNW will remain suitable with some low to moderate suitability changes. Likewise, a global study on *P. crassipes* using CLIMEX modelling predicts poleward range expansion in the Northern hemisphere; although, populations in the PNW may not persist, potentially dying off each winter (Kriticos and Brunel 2016). As with the terrestrial species, many studies of aquatic species do so at a global scale (e.g., Kriticos and Brunel 2016; Gillard et al. 2017), yet regional-scale modelling provides predictions on a scale that may be more effective for local land managers to target areas for increased management.

### Sources of uncertainty

To make predictions about the future habitat suitability of invasive plant species, we aimed to minimize the uncertainties associated with habitat suitability models (HSMs). The use of multiple algorithms, multiple GCMs, and multiple RCP scenarios, can account for some of the variability in modelled outputs, mitigating some of the associated uncertainty (Thuiller et al. 2019). Algorithm selection can be a large source of uncertainty, as each algorithm contains its own assumptions and limitations (Pearson et al. 2006). While all algorithms we employed make use of presence and pseudo-absence (or background) data, compared to methods that use presence-only data, the combination of regression and machine-learning techniques we employed all have the individual potential to produce equally valid representations of a given system (Araujo and New 2007). Although some finely adjusted single-algorithm models have been shown to perform better than ensemble models (Hao et al. 2020), several studies have found ensemble models perform as well or better than individual models (Marmion et al. 2009; Hao et al. 2019). Further studies are needed to validate the predictive accuracy of HSMs and, as such, there is no single ‘best’ method to produce consistently predictive results across any given taxa or application of use (Qiao et al. 2015).

HSMs are a useful tool to provide general predictions of potential expansions or contractions in the habitat suitability of invasive species; however, these methods contain assumptions and limitations. One assumption is that the species in question is at equilibrium in its environment, but this is often not the case when considering invasive species, which may not at the present time be found in the entire current potentially suitable habitat in the introduced range, and may never fully realize the current or future projected range of suitable habitat due to potential barriers to dispersal (Gallien et al. 2012; Barbet-Massin et al. 2018). Additionally, the use of invasive range data compared to global occurrences may result in an underestimation of potentially suitable habitat, as the species may occupy a somewhat different niche space in its invaded range compared to its native range (Barbet-Massin et al. 2018). Likewise, the current climate associated with species records locations used in HSMs may not reflect the current climate in new regions and/or with future climates in the same or new regions. To address this assumption, Multivariate Environmental Similarity Surfaces analyses can be used to assess the extent of extrapolation between current and future climates and the environmental similarity between regions (Elith et al. 2010). For the four study species, the MESS analysis shows that some caution may be necessary when assessing model predictions at high elevations at high latitudes (i.e., northern BC) or east of the Cascade Mountain range in the case of *P. officinarum*.

Second, due to data constraints, our models consider abiotic factors only, even though biotic factors, including species interactions and dispersal ability, may influence the amount of suitable habitat available. Likewise, due to unavailable future predictions for non-climatic environmental variables, such as future land use, our model contains the assumption of an un-changed non-climatic environment. Other studies have suggested that the influence of non-climatic variables is negligible in comparison to climatic changes (Sittaro et al. 2023); thus, we consider this assumption to have minimal influence on our models, although greater caution should be taken when considering species strongly influenced by anthropogenic factors.

Third, climate change may cause stochastic events, such as flooding or heat waves, which are difficult to account for within HSMs. Finally, for aquatic invasive species in particular, HSMs may benefit from the inclusion of water-specific variables such as flow rate, pH, or oxygen levels. On the other hand, climatic and anthropogenic variables have been most often used and contribute substantially to the suitability of freshwater aquatic plants (Rodríguez-Merino et al. 2018, Gillard et al. 2020, Ngarega et al. 2022). While these limitations or data constraints may result in models that tend towards over-prediction, this can often be more valuable when considering the distribution of invasive species (Jiménez-Valverde et al. 2011). Therefore, while the predicted impacts of climate change on a particular species as assessed by HSMs can only be considered an initial approximation (Beaumont et al. 2009), HSMs can still effectively project the geographic areas most likely to be invaded next, which is often the most vital information for land managers (Jiménez-Valverde et al. 2011; Barbet-Massin et al. 2018; Cordier et al. 2020).

### Implications for management strategies

Our results provide information necessary to focus management strategies targeting known invasive species that have not yet become established and suggest regional modelling should be considered in addition to global or continental models showing general trends. While risk assessments are critical to determine preventative measures, not all invasive species will be affected by climate change equally. Under all climate change scenarios, our models predict either the maintenance of moderate suitability or the potential increase in suitability for *G. lucidum* in most areas of the PNW. Current strategies to eradicate current populations, or prevent future establishment, should be continued as the species is not predicted to be deterred by climate change. Conversely, the current suitable habitat of *P. officinarum* is predicted to contract into higher elevations and some higher latitudes. While strategies should be put in place to prevent the spread of *P. officinarum* to higher elevations, management strategies focused on the most sensitive ecosystems may be adequate. The ability of *P. officinarum* to create dense mats and spread rapidly in local areas (Bishop and Davy 1994) still must be guarded against in more sensitive ecosystems even if the overall suitable habitat declines.

While terrestrial ecosystems have often been the focus, freshwater ecosystems are predicted to experience even greater pressure from invasive species under climate change (Havel et al. 2015). Species from tropical and subtropical native ranges, such as *P. crassipes*, show a greater potential for increased suitability in temperate regions under climate change. While our models show moderate suitability increases for *P. crassipes* in the PNW, further studies are necessary to determine whether *P. crassipes* is consistently able to overwinter. While the regions of moderate to high suitability may not be consistently suitable for the persistence of *P. crassipes* through the winter, our model suggests the target areas in which increased monitoring may be necessary to prevent the future establishment. Overall, *P. crassipes* has had detrimental impacts throughout its introduced range (Villamagna and Murphy 2010) and our results suggest the PNW has the potential for greater suitability under climate change scenarios. Alternatively, *B. umbellatus* is not predicted to increase in suitability along coastal regions, but habitat suitability may increase with climate change in regions that experience continental climates. Nevertheless, due to the variable nature of water body size and depth, localized coastal regions that experience more extreme conditions and are highly influenced by human activity may satisfy suitability requirements compared to surrounding areas. Furthermore, regions of current habitat suitability are predicted to remain suitable, suggesting current eradication plans should continue in order to prevent further spread of *B. umbellatus* in the PNW.

## Conclusions

The effects of climate change are being realized on regional scales throughout the world and, thus, are impacting the spread of recently introduced or previously unknown invasive species. While habitat suitability models have been used extensively and are necessary to assess over-arching global trends and patterns regarding species movement, our results at the regional scale show that invasive plant species are not impacted by climate change equally. The terrestrial species *G. lucidum* has the potential to expand northward, gaining suitable habitat, while *P. officinarum* will potentially lose much of its suitable habitat. Additionally, the aquatic species *B. umbellatus* will potentially gain moderately suitable habitat in the future, but only in inland regions. Conversely, *P. crassipes* with its tropical origins is currently limited to more coastal, mild climates and is not predicted to gain any substantial suitable habitat even by 2080. These terrestrial and aquatic species provide an initial sampling of differences seen between species as they respond to future climate scenarios, suggesting that not all invasive species will have increased negative effects under climate change. Furthermore, more significant changes in habitat suitability due to climate change were found when assessing the terrestrial species, compared to the aquatic species. Thus, our research provides a working template for the necessary modelling of additional species of concern on a regional scale. Species assessed individually at a localized scale provide models necessary for local land managers to develop targeted, preventative management strategies when coupled with their local invasive species risk assessments. This study provides valuable insight into the regional impact of climate change and human influences on aquatic and terrestrial invasive plant species and contributes to accessible predictive modelling for land managers and practitioners focusing on preventing the establishment of potentially detrimental invasive plant species.

### Supplementary Information

Below is the link to the electronic supplementary material.

Supplementary file1 (DOCX 1845 KB)

## Data Availability

The datasets generated during and/or analyzed during the current study are available in a GitHub repository, https://github.com/enikkel/PNW-Habitat-Suitability-Modelling.
