# Supplementary material for: Regional habitat suitability for aquatic and terrestrial invasive plant species may expand or contract with climate change
Source: Biol Invasions. 2023 Jul 28;25(12):3805–22. doi: 10.1007/s10530-023-03139-8 (PMC10579163; doi:10.1007/s10530-023-03139-8)
Supplement: Supplementary file 1 — Supplementary file1 (DOCX 1845 KB) [file 10530_2023_3139_MOESM1_ESM.docx]

Regional habitat suitability for aquatic and terrestrial invasive plant species may expand or contract with climate change; *Biological Invasions*; Emma Nikkel, David R. Clements, Delia Anderson, Jennifer L. Williams; [nikkel.emma@gmail.com](mailto:nikkel.emma@gmail.com)

**Electronic supplementary material**

**Appendix S1 – Data Sources.** Detailed methods for species record collection, cleaning protocol, and R code for correlation analysis and Biomod2 modelling is available in an open access repository, found at https://github.com/enikkel/PNW-Habitat-Suitability-Modelling.

Table S1.1. Species record data sources

| **Species** | **Data source and citation** |
| --- | --- |
| *Geranium lucidum* L. | GBIF. 2022. GBIF Occurrence. https://doi.org/10.15468/dl.g7xuqd Retrieved 08/19/2021. |
|  | EDDMapS. 2022. *Geranium lucidum* L. [Dataset]*.* Early Detection & Distribution Mapping System. The University of Georgia - Center for Invasive Species and Ecosystem Health. https://bugwoodcloud.org/eddmaps/shp/32876.zip Retrieved 06/29/2021. |
|  | IAPP. 2022. Invasive Alien Plant Program. http://maps.gov.bc.ca/ess/hm/iapp/ Retrieved 04/07/2022. |
| *Pilosella officinarum* Vaill. | GBIF. 2022. GBIF Occurrence https://doi.org/10.15468/dl.86h3gy Retrieved 08/19/2021. |
|  | EDDMapS. 2022. *Hieracium pilosella* L. [Dataset]*.* Early Detection & Distribution Mapping System. The University of Georgia - Center for Invasive Species and Ecosystem Health. https://bugwoodcloud.org/eddmaps/shp/32962.zip.  Retrieved 07/15/2021. |
|  | IAPP. 2022. Invasive Alien Plant Program. http://maps.gov.bc.ca/ess/hm/iapp/ Retrieved 04/07/2022. |
| **Species** | **Data source and citation** |
| *Butomus umbellatus* L. | GBIF. 2022. GBIF Occurrence https://doi.org/10.15468/dl.b7dtg3 Retrieved 08/19/2021. |
|  | EDDMapS. 2022. *Butmous umbellatus* L. [Dataset]*.* Early Detection & Distribution Mapping System. The University of Georgia - Center for Invasive Species and Ecosystem Health. https://bugwoodcloud.org/eddmaps/shp/32963.zip. Retrieved 07/15/2021. |
|  | IAPP. 2022. Invasive Alien Plant Program. http://maps.gov.bc.ca/ess/hm/iapp/ Retrieved 04/07/2022. |
| *Pontederia crassipes* Mart. | GBIF. 2022. GBIF Occurrence https://doi.org/10.15468/dl.m2fjgj Retrieved 08/19/2021. |
|  | EDDMapS. 2022. *Eichhornia crassipes* Mart.  [Dataset]*.* Early Detection & Distribution Mapping System. The University of Georgia - Center for Invasive Species and Ecosystem Health. https://bugwoodcloud.org/eddmaps/shp/32880.zip. Retrieved 06/29/2021. |
|  | IAPP. 2022. Invasive Alien Plant Program. http://maps.gov.bc.ca/ess/hm/iapp/ Retrieved 04/07/2022. |

Table S1.2. Environmental data sources

| **Dataset** | **Data source** | **Access Date** |
| --- | --- | --- |
| ClimateNA | AdaptWest Project (2021) Gridded current and projected climate data for North America at 1km resolution, generated using the ClimateNA v7.01 software. http://adaptwest.databasin.org. | May 30, 2022 |
| Global Human Influence Index | Wildlife Conservation Society - WCS, and Center for International Earth Science Information Network - CIESIN - Columbia University (2005) Last of the Wild Project, Version 2, 2005 (LWP-2): Global Human Influence Index (HII) Dataset (Geographic). Palisades, NY: NASA Socioeconomic Data and Applications Center (SEDAC). https://doi.org/10.7927/H4BP00QC. | December 6, 2021 |

Table S1.3. General circulation model sources

| **Model** | **Institution** | **Citation** |
| --- | --- | --- |
| MRI-ESM2.0 | Meteorological Research Institute (Japan) | Yukimoto S, Kawai H, Koshiro T, Oshima N, Yoshida K, Urakawa S, et al. (2019) The meteorological research institute Earth system model version 2.0, MRI-ESM2.0: description and basic evaluation of the physical component. Journal of the Meteorological Society of Japan 97:931–965. https://doi.org/10.2151/jmsj.2019-051 |
| UKESMI1.0-LL | Met Office Hadley Centre and Natural Environment Research Council (UK) | Sellar AA, Jones CG, Mulcahy JP, Tang Y, Yool A, Wiltshire A, et al. (2019) UKESM1: description andevaluation of the U.K. earth system model. Journal of Advances in Modeling Earth Systems 11:4513–4558. https://doi.org/10.1029/2019MS001739 |
| MPI-ESM1.2-HR | Max Planck Institute for Meteorology (Germany) | Müller WA, Jungclaus JH, Mauritsen T, Baehr J, Bittner M, Budich R, et al. (2018) A higher-resolution version of the MaxPlanck Institute earth system model (MPI-ESM1.2-HR). Journal of Advances in Modeling Earth Systems 10:1383–1413. http://doi.org/10.1029/2017MS001217 |

**Appendix S2 – Variable selection.**

Table S2.1. Environmental variables and acronyms.

| **Variable** | **Acronym** |
| --- | --- |
| Mean annual temperature (°C) | MAT |
| Mean warmest month temperature (°C) | MWMT |
| Mean coldest month temperature (°C) | MCMT |
| Temperature difference between MWMT and MCMT, or continentality (°C) | TD |
| Mean annual precipitation (mm) | MAP |
| May to September precipitation (mm) | MSP |
| Annual heat-moisture index (MAT+10)/(MAP/1000)) | AHM |
| Summer heat-moisture index ((MWMT)/(MSP/1000)) | SHM |
| Degree-days below 0°C (chilling degree-days) | DD_0 |
| Degree-days above 5°C (growing degree-days) | DD5 |
| Degree-days below 18°C (heating degree-days) | DD_18 |
| Degree-days above 18°C (cooling degree-days) | DD18 |
| The number of frost-free days | NFFD |
| Frost-free period (FFP) | FFP |
| Day of the year on which FFP begins | bFFP |
| Day of the year on which FFP ends | eFFP |
| Precipitation as snow (mm) between August in previous year and July in current year | PAS |
| Extreme minimum temperature over 30 years | EMT |
| Extreme maximum temperature over 30 years | EXT |
| Hargreaves reference evaporation (mm) | Eref |
| Hargreaves climatic moisture deficit (mm) | CMD |
| Mean annual relative humidity (%) | RH |
| Hogg’s climate moisture index (mm) | CMI |
| Degree-days above 10°C and below 40°C | DD1040 |
| Winter mean temperature (°C) (Dec – Feb) | Tave_wt |
| Spring mean temperature (°C) (Mar – May) | Tave_sp |
| Summer mean temperature (°C) (Jun – Aug) | Tave_sm |
| Autumn mean temperature (°C) (Sept – Nov) | Tave_at |
| Winter precipitation (mm) (Dec – Feb) | PPT_wt |
| Spring precipitation (mm) (Mar – May) | PPT_sp |
| **Variable** | **Acronym** |
| Summer precipitation (mm) (Jun – Aug) | PPT_sm |
| Autumn precipitation (mm) (Sept – Nov) | PPT_at |
| Human influence index | HII |

Table S2.2. *Geranium lucidum* variable VIF scores below the threshold of 5.

| **Environmental variable** | **VIF score** |
| --- | --- |
| Degree-days above 18°C | 2.68 |
| Summer precipitation | 2.21 |
| Winter precipitation | 2.13 |
| Extreme maximum temperature over 30 years | 1.84 |
| Degree-days below 0°C | 1.77 |
| Human influence index | 1.75 |
| Summer heat moisture index | 1.56 |

Table S2.3. *Pilosella officinarum* variable VIF scores below the threshold of 5.

| **Environmental variable** | **VIF score** |
| --- | --- |
| Extreme maximum temperature over 30 years | 3.98 |
| Day of the year on which the frost-free period begins | 3.50 |
| Relative humidity | 3.47 |
| Degree-days below 0°C | 2.36 |
| Annual heat moisture index | 2.08 |
| Human influence index | 1.42 |
| May to September precipitation | 1.40 |

Table S2.4. *Butomus umbellatus* variable VIF scores below the threshold of 5.

| **Environmental variable** | **VIF score** |
| --- | --- |
| Degree-days below 0°C | 4.86 |
| Winter precipitation | 4.15 |
| Precipitation as snow | 3.37 |
| Relative humidity | 2.53 |
| Extreme maximum temperature over 30 years | 2.43 |
| Summer precipitation | 2.39 |
| Human influence index | 1.16 |

Table S2.5. *Pontederia crassipes* variable VIF scores below the threshold of 5.

| **Environmental variable** | **VIF score** |
| --- | --- |
| Number of frost-free days | 3.41 |
| Precipitation as snow | 3.18 |
| Autumn precipitation | 2.56 |
| Relative humidity | 1.99 |
| Summer heat moisture index | 1.71 |
| Extreme maximum temperature over 30 years | 1.63 |
| Winter precipitation | 1.45 |
| Human influence index | 1.07 |

**Appendix S3 – Variable importance and response of the species to environmental variables.** The weighted mean (shown in purple), rather than the mean (shown in blue), of all included models was used to produce the final current climate ensemble model, according to Marmion et al. (2009).


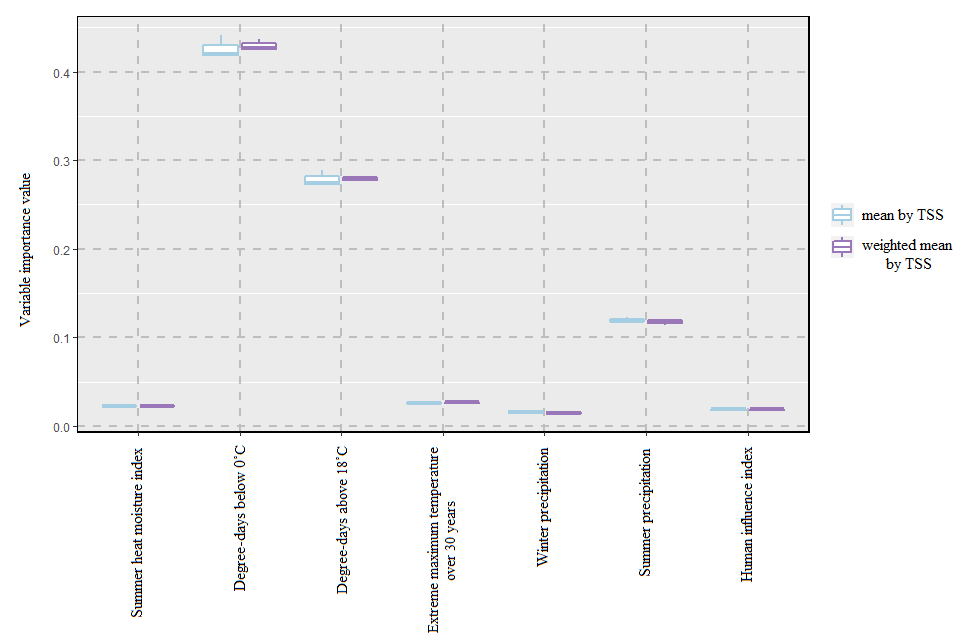


Figure S3.1. Variable importance of variables selected for the *Geranium lucidum* model, according to the variable importance procedure in Biomod2 (Thuiller et al. 2009).


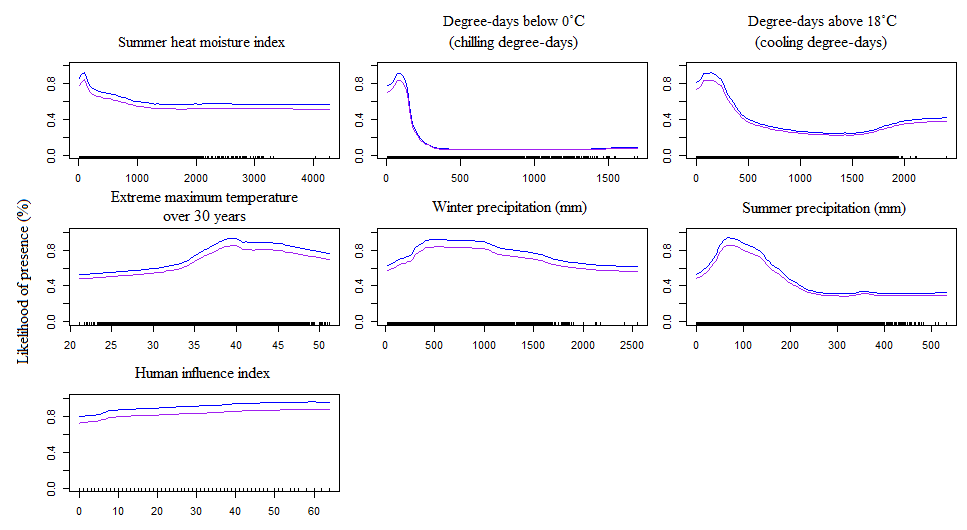


Figure S3.2. Response of *Geranium lucidum* to the selected environmental predictor variables. Blue lines indicate mean values, while purple lines indicate the weighted mean values.


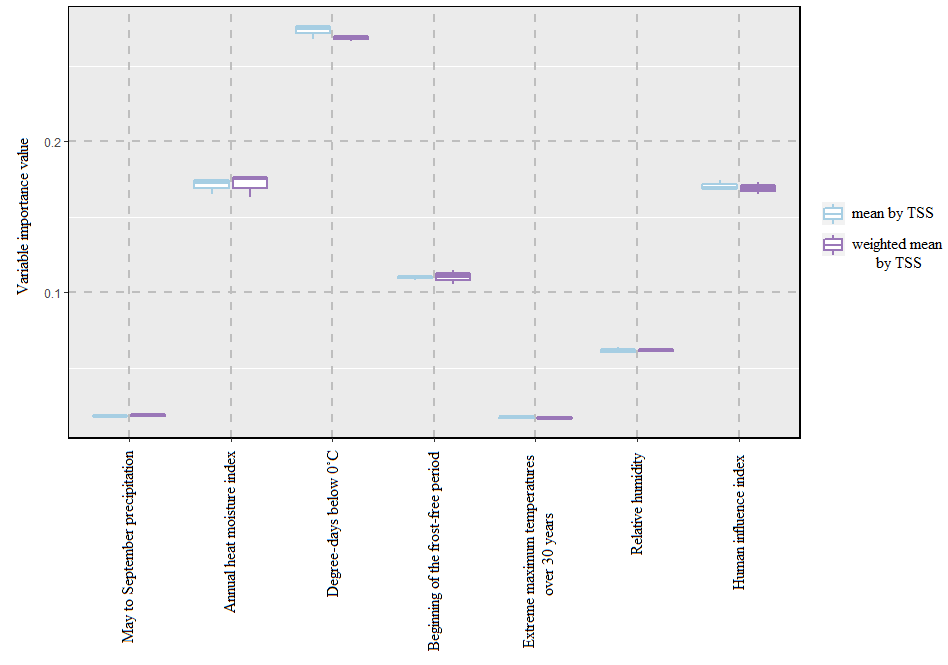


Figure S3.3. Variable importance of variables selected for the *Pilosella officinarum* model, according to the variable importance procedure in Biomod2 (Thuiller et al. 2009).


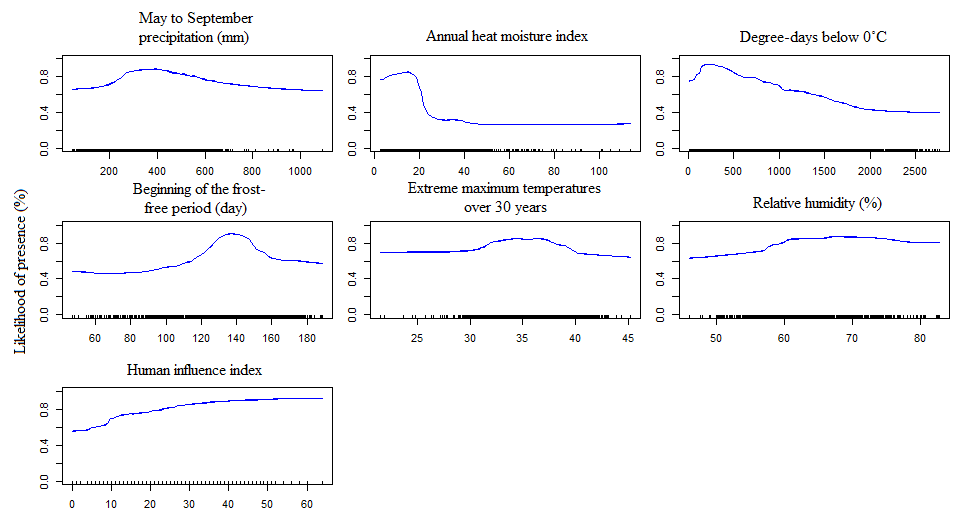


Figure S3.4. Response of *Pilosella officinarum* to the selected environmental predictor variables. Both the weighted mean and mean resulted in the same values and, thus, are shown as one line.


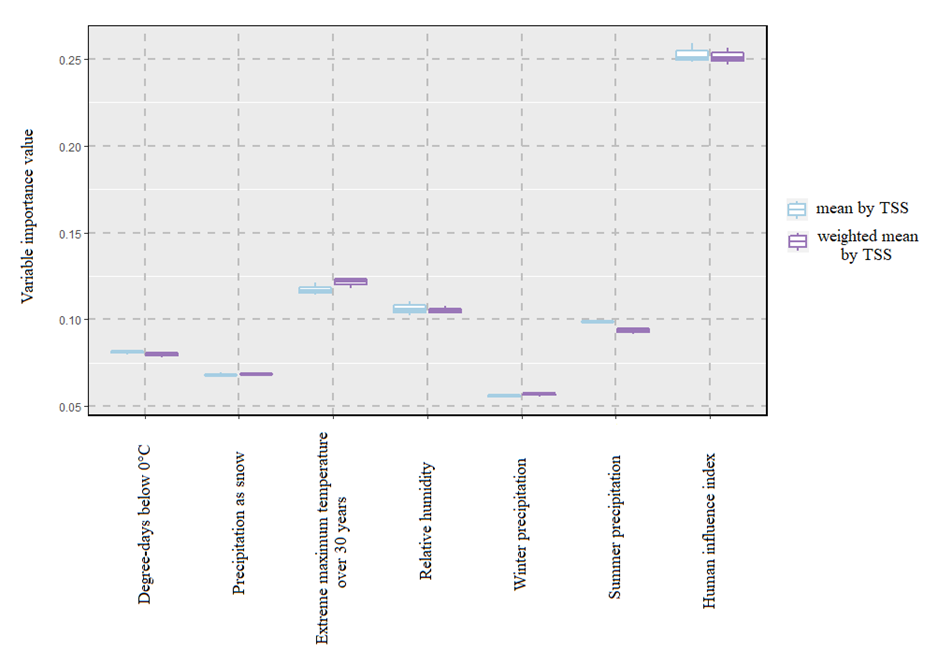


Figure S3.5. Variable importance of variables selected for the *Butomus umbellatus* model, according to the variable importance procedure in Biomod2 (Thuiller et al. 2009).


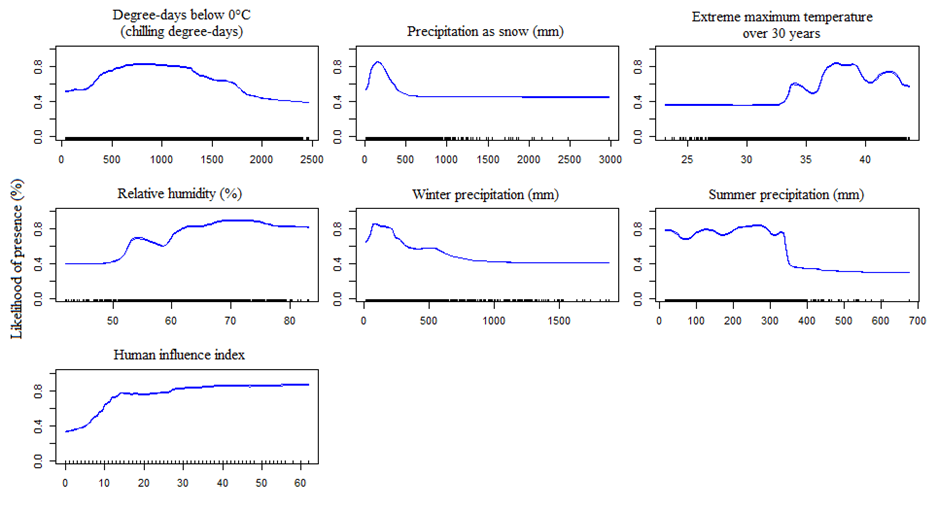


Figure S3.6. Response curve of *Butomus umbellatus* to the selected environmental predictor variables.


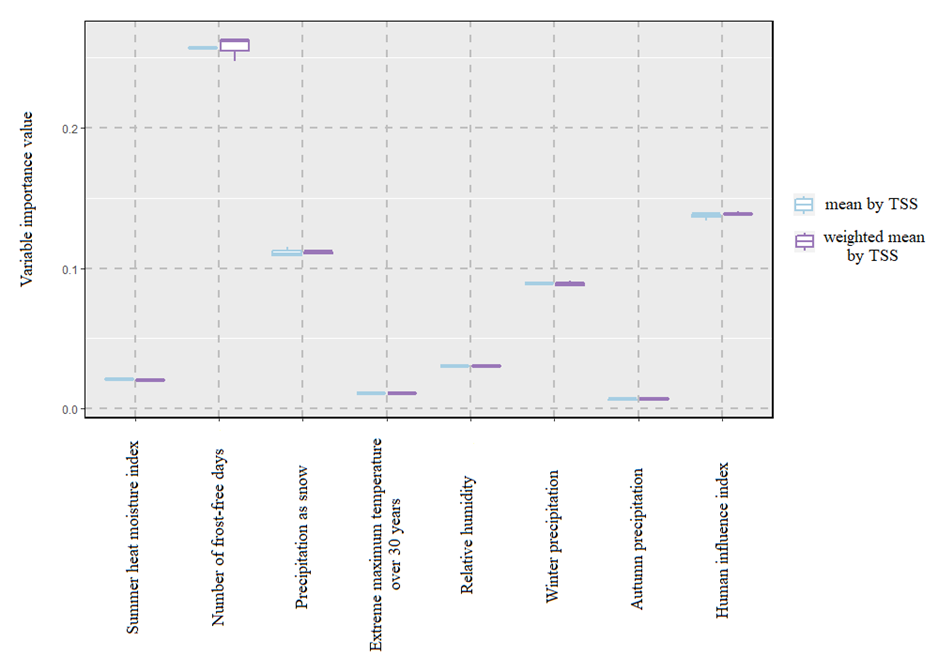


Figure S3.7. Variable importance of variables selected for the *Pontederia crassipes* model, according to the variable importance procedure in Biomod2 (Thuiller et al. 2009).


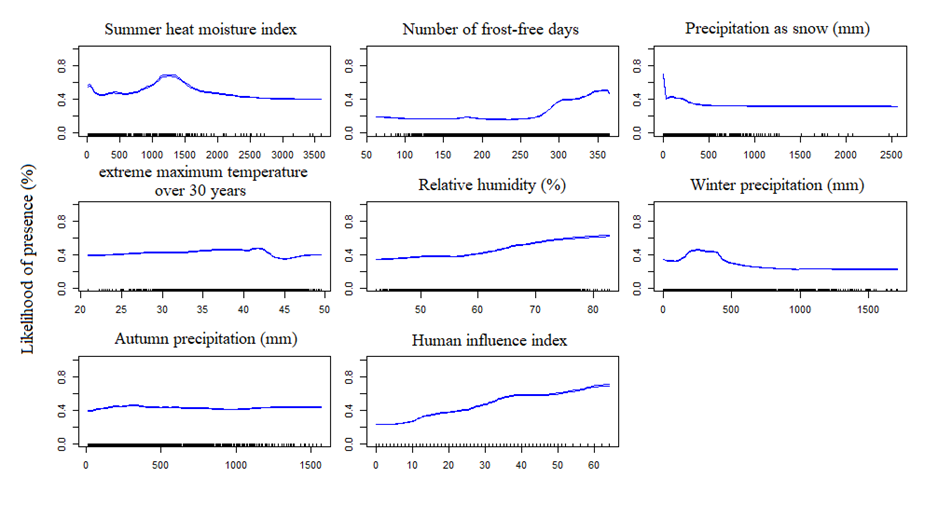


Figure S3.8. Response curve of *Pontederia crassipes* to the selected environmental predictor variables.

**Appendix S4 – Multivariate environmental similarity surfaces analyses.**


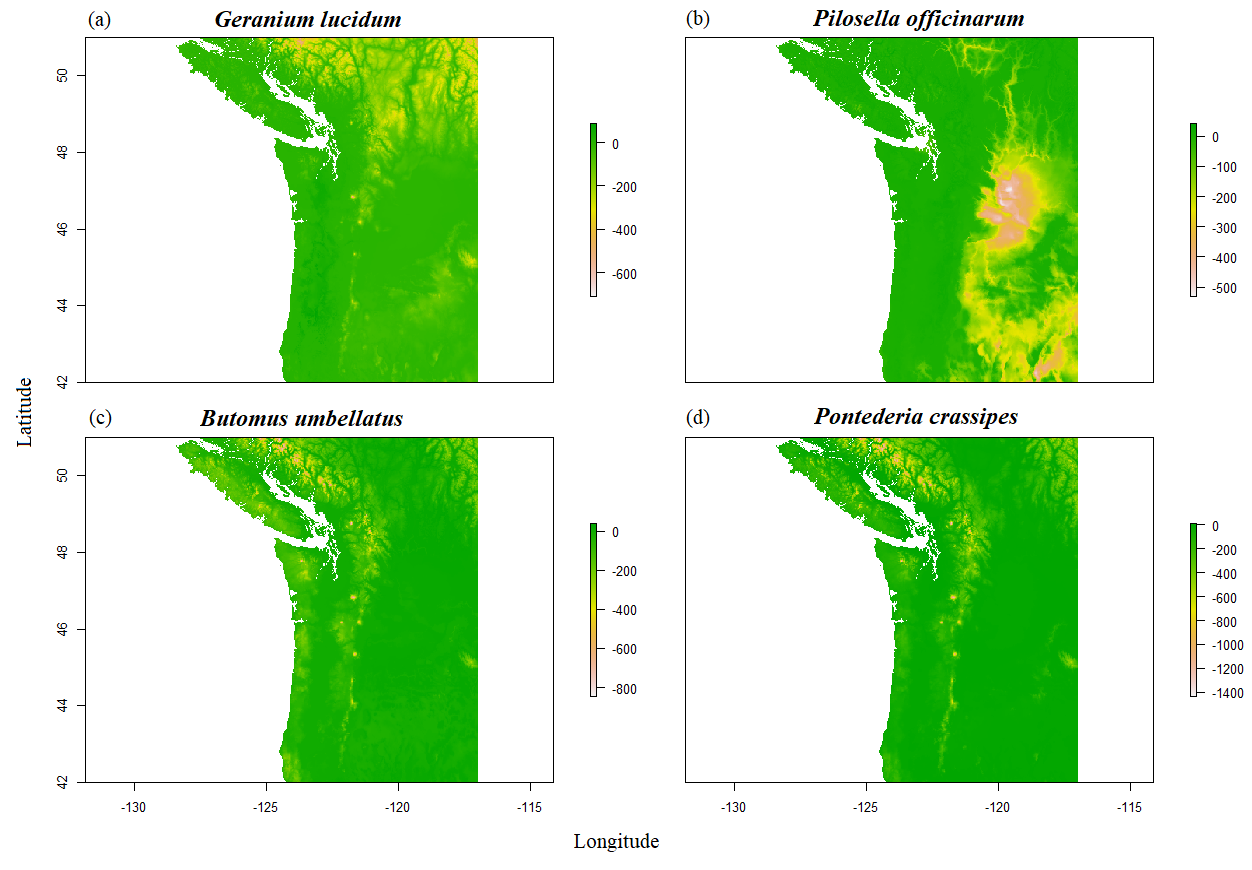


Figure S4.1. Multivariate environmental similarity surfaces analysis for (a) *Geranium lucidum*, (b) *Pilosella officinarum*, (c) *Butomus umbellatus*, and (d) *Pontederia crassipes*. The analysis shows the level of similarity between the current climate at the species record locations and the current climate of the Pacific Northwest, North America. Higher values indicate greater similarity between climates.

**Appendix S5 – Future habitat suitability maps.**


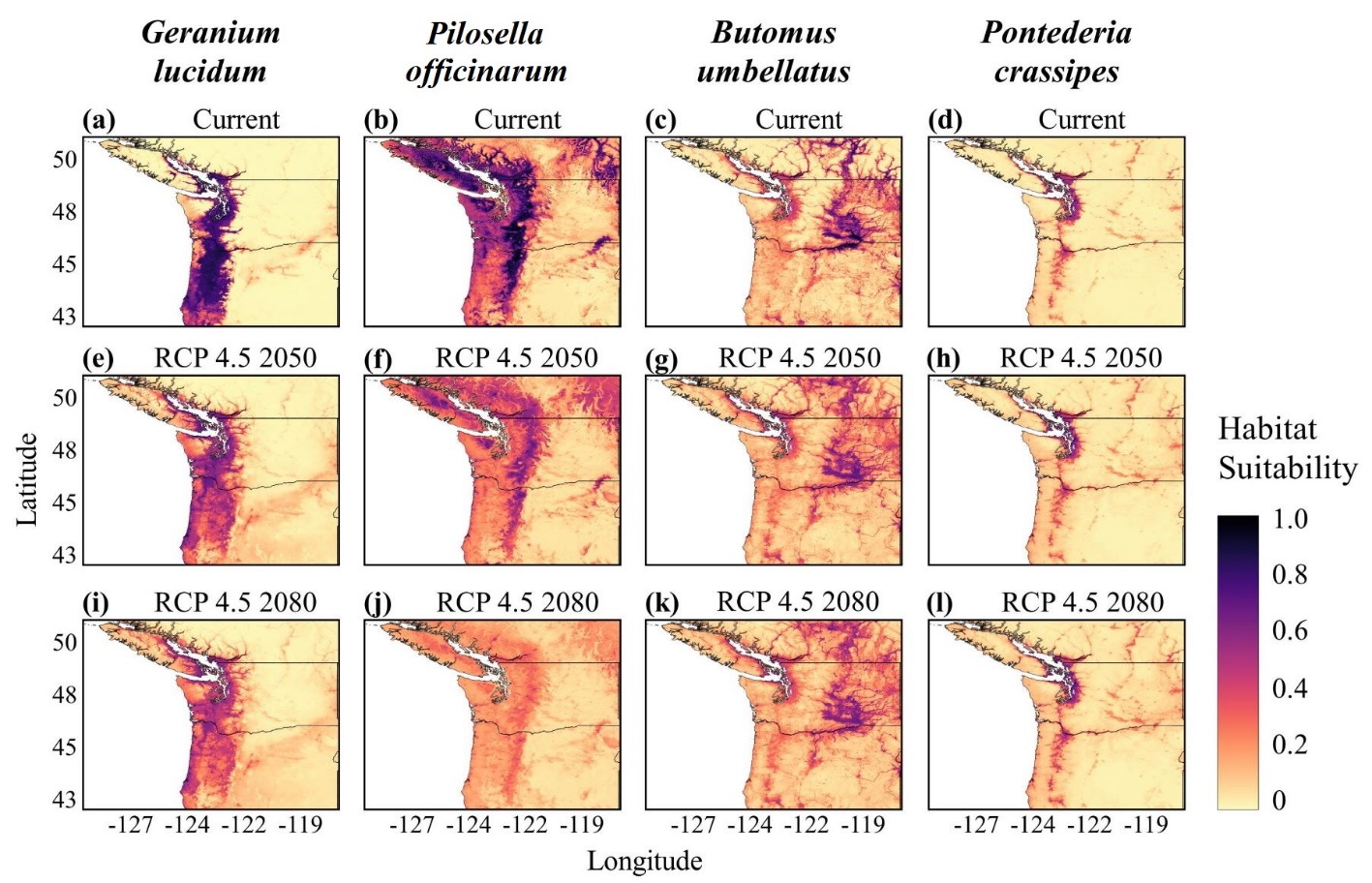


Figure S5.1. Current and projected future habitat suitability of the PNW for *Geranium lucidum*, *Pilosella officinarum, Butomus umbellatus,* and *Pontederia crassipes*, according to RCP scenario 4.5. (a - d) Current potential habitat suitability for *Geranium lucidum, Pilosella officinarum, Butomus umbellatus,* and *Pontederia crassipes*, respectively; (e - h) potential habitat suitability under climate scenario RCP 4.5 for the 2050s for *Geranium lucidum, Pilosella officinarum, Butomus umbellatus,* and *Pontederia crassipes*, respectively; (i - l) potential habitat suitability under climate scenario RCP 4.5 for the 2080s for *Geranium lucidum, Pilosella officinarum, Butomus crassipes,* and *Pontederia crassipes*, respectively.


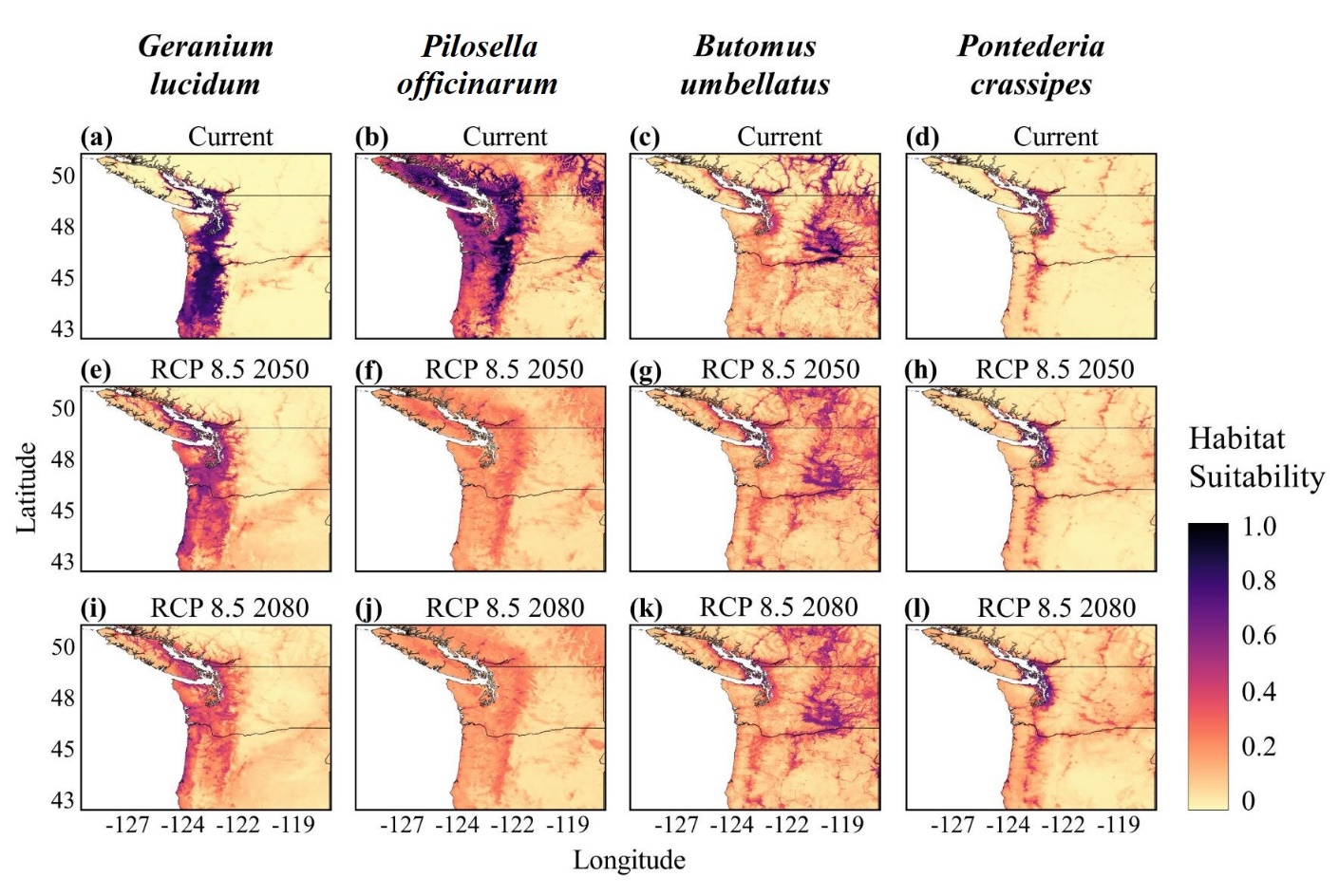


Figure S5.2. Current and projected future habitat suitability of the PNW for *Geranium lucidum*, *Pilosella officinarum, Butomus umbellatus,* and *Pontederia crassipes*, according to RCP scenario 8.5. (a - d) Current potential habitat suitability for *Geranium lucidum, Pilosella officinarum, Butomus umbellatus,* and *Pontederia crassipes*, respectively; (e - h) potential habitat suitability under climate scenario RCP 8.5 for the 2050s for *Geranium lucidum, Pilosella officinarum, Butomus umbellatus,* and *Pontederia crassipes*, respectively; (i - l) potential habitat suitability under climate scenario RCP 8.5 for the 2080s for *Geranium lucidum, Pilosella officinarum, Butomus umbellatus,* and *Pontederia crassipes*, respectively.


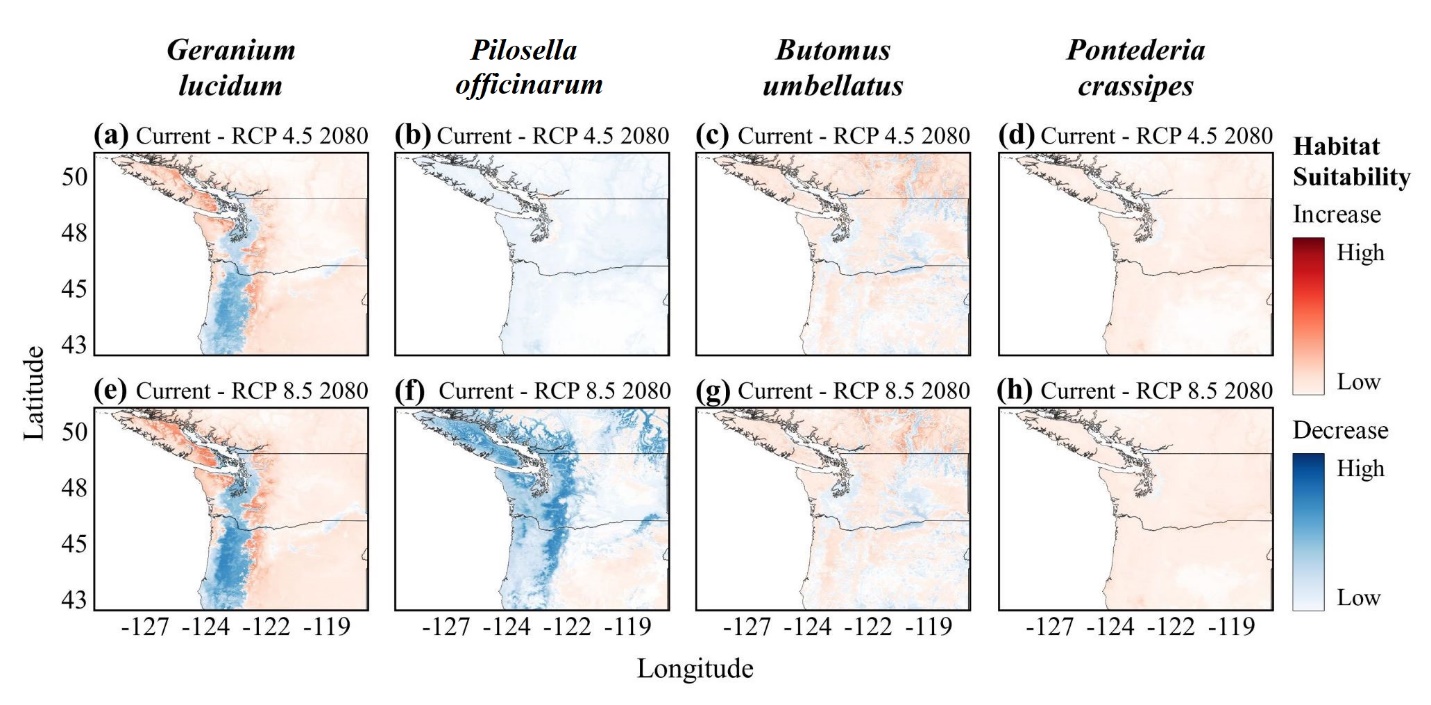


Figure S5.3. Potential expansion or contraction of habitat suitability under future climate scenarios by 2080. (a – d) Increases and decreases in the predicted habitat suitability of *Geranium lucidum*, *Pilosella officinarum*, *Butomus umbellatus*, and *Pontederia crassipes*, respectively, under climate scenario 4.5 by 2080; (e – h) increases and decreases in the predicted habitat suitability of *Geranium lucidum*, *Pilosella officinarum*, *Butomus umbellatus*, and *Pontederia crassipes*, respectively, under climate scenario 8.5 by 2080.

Regional habitat suitability for aquatic and terrestrial invasive plant species may expand or contract with climate change

– ODMAP Protocol –

Emma Nikkel, David R. Clements, Delia Anderson, Jennifer L. Williams

2023-04-11

## Overview

#### Authorship

Contact : [nikkel.emma@gmail.com](mailto:nikkel.emma@gmail.com)

<Study link>

#### Model objective

Model objective: Forecast and transfer

Target output: suitable vs. unsuitable habitat, change under future conditions

#### Focal Taxon

Focal Taxon: Invasive plants

#### Location

Location: Pacific Northwest, North America

#### Scale of Analysis

<Spatial extent>

Spatial resolution: 1

Temporal extent: Current (1981-2010), 2050, 2080

Boundary: natural, rectangle

#### Biodiversity data

Observation type: citizen science, range map

Response data type: point occurrence, presence-only

#### Predictors

Predictor types: climatic, human influences

#### Hypotheses

Hypotheses: Using habitat suitability models, we aimed (1) to establish the current potential habitat suitability of four relatively new invasive plant species to the PNW region of North America, assessing the relative contributions of climate and human influence, and (2) to predict the future habitat suitability for these species in the PNW, assessing the potential expansion or contraction of the distribution of these species with climate change, and contrasting across species from different habitat types.

#### Assumptions

Model assumptions: We assumed that the species are at pseudo-equilibrium with the environment. Additionally we assume that relevant ecological drivers (or proxies) of species distributions are included, sampling is adequate and representative with biases being accounted for, and that model predictions are not extrapolating beyond known climate (as shown through MESS analyses).

#### Algorithms

Modelling techniques: glm, mars, brt, ann, gam, randomForest

Model complexity: Default settings were used for all model algorithms.

Model averaging: We combined all model algorithms to create an ensemble model.

#### Workflow

Model workflow: Correlation analyses (using VIF) were performed and highly-correlated variables removed. Variable importance was assessed using the 'variable importance' function from the Biomod2 package. This procedure was repeated three times for each variable, finding the mean correlation coefficient over all cross-validation runs, resulting in a ranking of variable importance for each model. For each individual model and the final ensemble model, we evaluated the response of the species to environmental predictor variables with the evaluation strip method (Elith et al. 2005), to assess how each variable contributed to the model. Six model algorithms were used (GLM, MARS, BRT, ANN, GAM, RF). Models with a TSS > 0.7 were included in the ensemble model. To produce the final current climate ensemble model, we used the mean weighted by each model’s TSS value. Overall, a total of 300 projections of habitat suitability (6 modelling algorithms x 10 pseudo-absence runs x 5 cross-validation runs) were created for each species. One current climate habitat suitability map and three future climate habitat suitability maps per year (2050 and 2080) and per scenario (RCP 4.5, 7.0 and 8.5) were produced per species, after future habitat suitability maps were averaged per year and per scenario.

#### Software

Software: Analyses were conducted using R version 4.1.1 (R Core Team 2021) and packages: ‘CoordinateCleaner’ version 2.0.18 (Zizka et al 2019), ‘dplyr’ version 1.0.6 (Wickham et al. 2021), and ‘biomod2’ version 3.5.1 (Thuiller et al. 2009).

Code availability: Code files available through GitHub at <https://github.com/enikkel/PNW-Habitat-Suitability-Modelling>.

Data availability: Data files available through GitHub at <https://github.com/enikkel/PNW-Habitat-Suitability-Modelling>.

## Data

#### Biodiversity data

Taxon names: Geranium lucidum, Pilosella officinarum, Butomus umbellatus, Pontederia crassipes

<Taxonomic reference system>

Ecological level: species

Data sources: Species data gathered from GBIF, EDDMapS, and IAPP websites. References and accession dates are listed in the Supplemental Materials.

Sampling design: Records collected between 1981-2010.

Sample size: Geranium lucidum: 611 Pilosella officinarum: 241 Butomus umbellatus: 524 Pontederia crassipes: 654

Clipping: North America

Scaling: Records kept if spatial uncertainty was <1000m; only 1 record retained per square km.

Cleaning: Data cleaning included: records with inconsistent information removed, duplicate records removed, country centroid records removed.

<Absence data>

Background data: Pseudo-absence data was derived from random point selection within a spatial zone around presence records. The extent of the spatial zone was individually determined for each species using the methods described by VanDerWal et al. (2009). The number of pseudo-absence records used was equal to the number of presence records for each species.

#### Data partitioning

Training data: We randomly selected 70% of data to be used for training.

Validation data: We randomly selected 30% of data to be used for model testing/validation.

#### Predictor variables

Predictor variables: Geranium lucidum: Degree-days above 18°C, Summer precipitation, Winter precipitation, Extreme maximum temperature over 30 years, Degree-days below 0°C, Summer heat moisture index, Human influence index Pilosella officinarum: Extreme maximum temperature over 30 years, Day of the year on which the frost-free period begins, Relative humidity, Degree-days below 0°C, Annual heat moisture index, May to September precipitation, Human influence index Butomus umbellatus: Degree-days below 0°C, Winter precipitation, Precipitation as snow, Relative humidity, Extreme maximum temperature over 30 years, Summer precipitation, Human influence index Pontederia crassipes: Number of frost-free days, Precipitation as snow, Autumn precipitation, Relative humidity, Summer heat moisture index, Extreme maximum temperature over 30 years, Winter precipitation, Human influence index

Data sources: Bioclimatic variables: ClimateNA <https://adaptwest.databasin.org/pages/adaptwest-climatena/>, Accessed: 08/17/2021 Human Influence Index: SEDAC <https://sedac.ciesin.columbia.edu/data/set/wildareas-v2-human-influence-index-geographic/data-download>, Accessed 08/17/2021

Spatial extent: -180, -40, 10, 90 (xmin, xmax, ymin, ymax)

Spatial resolution: 1 km

Coordinate reference system: proj4 string

<Temporal extent>

#### Transfer data

<Data sources>

<Spatial extent>

<Spatial resolution>

<Temporal extent>

<Models and scenarios>

<Quantification of Novelty>

## Model

#### Multicollinearity

Multicollinearity: To identify collinearity between variables, correlations between all 34 variables were assessed by calculating a variance inflation factor (VIF) for each variable (R package ‘usdm’ version 1.1.18; Naimi et al. 2014). Variables were retained for the HSMs if they had a VIF of less than 5.

#### Model settings

glm: family ('Biomod2' package default GLM settings were used according to version 3.5.1)

mars: formula ('Biomod2' package default MARS settings were used according to version 3.5.1)

brt: formula ('Biomod2' package default BRT(GBM) settings were used according to version 3.5.1)

ann: formula ('Biomod2' package default ANN settings were used according to version 3.5.1)

gam: family ('Biomod2' package default GAM settings were used according to version 3.5.1)

randomForest: ntree ('Biomod2' package default RF settings were used according to version 3.5.1)

<Model settings (extrapolation)>

#### Model estimates

<Coefficients>

Variable importance: We assessed contributions of each variable to each species’ model through the ‘variable importance’ procedure in the ‘biomod2’ package (Thuiller et al. 2009). This procedure was repeated three times for each variable, finding the mean correlation coefficient over all cross-validation runs, resulting in a ranking of variable importance for each model.

#### Model selection - model averaging - ensembles

Model ensembles: Models with a TSS > 0.7 were included in the ensemble model. To produce the final current climate ensemble model, we used the mean weighted by each model’s TSS value.

#### Analysis and Correction of non-independence

<Spatial autocorrelation>

#### Threshold selection

<Threshold selection>

## Assessment

#### Performance statistics

<Performance on training data>

Performance on validation data: AUC, TSS

<Performance on test data>

#### Plausibility check

Response shapes: For each individual model and the final ensemble model, we evaluated the response of the species to environmental predictor variables with the evaluation strip method (Elith et al. 2005). These are available in the Supplemental Materials.

Expert judgement: One current climate habitat suitability map and three future climate habitat suitability maps per year (2050 and 2080) and per scenario (RCP 4.5, 7.0 and 8.5) were produced per species, after future habitat suitability maps were averaged per year and per scenario. These maps contain continuous probabilities of species occurrence, transformed into integers from 0 to 1000 by the biomod2 functions.

## Prediction

#### Prediction output

Prediction unit: Predictions of relative probability of presence expressed on a continuous scale.

#### Uncertainty quantification

Algorithmic uncertainty: Six algorithms were used and models with TSS values > 0.7 were averaged into an ensemble model.

Scenario uncertainty: The top three general circulation models (GCMs) for Western North American region were used to account for the spatial variation in climate change responses.

Novel environments: To assess the extent of extrapolation and environmental similarity between the climates where the species records were located and the PNW study region, we performed a Multivariate Environmental Similarity Surfaces (MESS) analysis.
